# Effect of in vitro cultivation on human gut microbiota composition using 16S rDNA amplicon sequencing and metabolomics approach

**DOI:** 10.1038/s41598-023-29637-2

**Published:** 2023-02-21

**Authors:** Paulina Średnicka, Marek Łukasz Roszko, Dominik Popowski, Monika Kowalczyk, Michał Wójcicki, Paulina Emanowicz, Magdalena Szczepańska, Danuta Kotyrba, Edyta Juszczuk-Kubiak

**Affiliations:** 1 Laboratory of Biotechnology and Molecular Engineering, Department of Microbiology, Prof. Wacław Dąbrowski Institute of Agricultural and Food Biotechnology – State Research Institute, Rakowiecka 36 Street, 02-532 Warsaw, Poland; 2 Department of Food Safety and Chemical Analysis, Prof. Wacław Dąbrowski Institute of Agricultural and Food Biotechnology – State Research Institute, Rakowiecka 36 Street, 02-532 Warsaw, Poland; 3 Microbiota Lab, Department of Pharmacognosy and Molecular Basis of Phytotherapy, Medical University of Warsaw, Banacha 1 Street, 02-097 Warsaw, Poland; 4 Department of Research, Scientific Information and Marketing Coordination, Prof. Wacław Dąbrowski Institute of Agricultural and Food Biotechnology – State Research Institute, Rakowiecka 36 Street, 02-532 Warsaw, Poland

**Keywords:** Bioinformatics, Genetic techniques, Genomic analysis, Metabolomics, Microbiology techniques, Sequencing, Microbial genetics, Microbiome

## Abstract

Gut microbiota (GM) plays many key functions and helps maintain the host’s health. Consequently, the development of GM cultivation under in vitro stimulating physiological conditions has gained extreme interest in different fields. In this study, we evaluated the impact of four culture media: Gut Microbiota Medium (GMM), Schaedler Broth (SM), Fermentation Medium (FM), and Carbohydrate Free Basal Medium (CFBM) on preserving the biodiversity and metabolic activity of human GM in batch in vitro cultures using PMA treatment coupled with 16S rDNA sequencing (PMA-seq) and LC-HR-MS/MS untargeted metabolomics supplemented with GC–MS SCFA profiling. Before the experiments, we determined the possibility of using the pooled faecal samples (MIX) from healthy donors (n = 15) as inoculum to reduce the number of variables and ensure the reproducibility of in vitro cultivation tests. Results showed the suitability of pooling faecal samples for in vitro cultivation study. Non-cultured MIX inoculum was characterized by higher *α*-diversity (Shannon effective count, and Effective microbial richness) compared to inocula from individual donors. After 24 h of cultivation, a significant effect of culture media composition on GM taxonomic and metabolomic profiles was observed. The SM and GMM had the highest *α*-diversity (Shannon effective count). The highest number of core ASVs (125) shared with non-cultured MIX inoculum and total SCFAs production was observed in the SM. These results might contribute to the development of standardized protocols for human GM in vitro cultivation by preventing methodological bias in the data.

## Introduction

The large intestine is inhabited by a vast number of bacteria, fungi, archaea, and viruses, making it the largest micro-ecosystem in the human body^1^. These microorganisms constantly interact with each other and with the host, playing an important role in maintaining human health^1,2^. The physiological functions of the GM include the maturation of the immune system^3^, energy harvest^4^, protection against pathogens^5^, gut barrier integrity maintenance^6^, enteroendocrine appetite signaling^7^ and others. The symbiotic balance state of GM can be easily disturbed by numerous endogenous and exogenous factors leading to dysbiosis, which may have adverse health outcomes not only in the gastrointestinal system but also in the neurological^8^, endocrine^9^, cardiovascular^10^ and immune system^11^. The role of GM contribution in the pathogenesis of human diseases is still not fully understood and requires further investigation to describe the molecular mechanisms and modes of action responsible for these processes^1,12^.

Currently, there is a need to optimize methods of in vitro culturing the human GM to investigate and understand its link with the pathogenesis of various diseases and food-induced alterations but also in the comprehensive evaluation of environmental pollutants toxicity, including endocrine disrupting chemicals (EDCs) as a major source of food contaminants and human exposure^13–15^. The use of in vitro models can provide a time- and cost-effective insight into microbiome responses and can deliver preliminary data for justifying further in vivo research^14^. Currently used in vitro models for the cultivation of GM include simple batch fermentation models, continuous fermentation models, and advanced culture systems to study microbiota-host interaction^14,16^.

Dominant members of GM are fastidious anaerobic microorganisms that require rich media and oxygen-free handling on every step from donation to cultivation^17^. The choice of cultivation medium is a crucial factor affecting the survivability of various GM species, and thus causes shifts in taxonomic profiles as well as changes in functional and metabolic levels manifested via the short-chain fatty acid (SCFAs) concentrations, and vitamins and small metabolites production^18,19^. To date, numerous commercially available and newly composed media have been used in various GM in vitro cultivation studies^20–23^, but many gut bacteria are still non-culturable due to the inability to simulate their natural environment in vitro^24^. Moreover, the distinction between live and dead bacteria is especially important when using sequencing methods to study the GM community. Next-generation sequencing (NGS) analyzes total DNA in samples containing viable bacteria, non-viable bacteria, and cell-free DNA in the environment leading to an overestimation of some taxa in the complex microbial community^25^. Recently, it has been reported that propidium monoazide (PMA), a DNA chelating agent that is excluded by an undamaged bacterial membrane, can be used to test the viability of the cells to reduce bias associated with 16S rDNA sequencing of various environmental samples^26^ including human stool to evaluate faecal microbiota transplantation protocols^27,28^. However, up to date, no studies on determining the impact of various types of media on human GM in vitro cultivation have been reported, where the influence of the application of PMA-sequencing (PMA-seq) was assessed. It should be noted that studying microbiome function at the individual level is challenging due to the high diversity of GM among humans^29^. Recently, several studies have focused on the feasibility of using the faecal pooling procedure to optimize a low-cost, large-scale sampling methodology, which could be utilized in various analyses of the human microbiome function^30,31^.

The main objective of this study was to evaluate the effect of the four commonly used microbial culture media to preserve the biodiversity and metabolic activity of the human GM in an in vitro batch-cultivation model. In the first part of this study, we determined the possibility of using the pooled faecal samples (MIX) from healthy donors (n = 15) as inoculum to reduce the number of variables and ensure the reproducibility of tests. Next, the PMA treatment to differentiate the viable microbes from non-viable GM community members coupled with targeted 16S rDNA sequencing (PMA-seq), and LC-HR-MS/MS untargeted metabolomics supplemented with GC–MS SCFA profiling was applied to examine the impact of cultivation media on the GM community and metabolomic profiles.

## Materials and methods

### Materials

Media used in this study included: Gut Microbiota Medium (GMM) without agar^32^, Schaedler Broth (SM) (Graso Biotech, Poland), Fermentation Medium (FM)^33^, and Carbohydrate Free Basal Medium (CFBM)^34^. All media were prepared following the manufacturer’s protocols. High purity SCFAs standards for gas chromatography (GC–MS) analysis, including acetic acid, propionic acid, butyric acid, isobutyric acid, isovaleric acid, and valeric acid, were purchased from Sigma Aldrich (St Louis, MO, USA). Human fresh faecal samples were collected from healthy volunteers (n = 15) recruited in Poland. All donors provided written informed consent prior to participating in the study; samples were anonymized and treated according to medical ethical guidelines^35^.

### Methods

#### Stool collection and preparation of the human GM inoculum

The human GM inoculum was prepared in the Laboratory of Biotechnology and Molecular Engineering in the Institute of Agricultural and Food Biotechnology—State Research Institute (IAFB-SRI) following the protocol approved by the Research Ethics Committee with Human Participation at the Warsaw University of Life Sciences (#23/2021). Healthy donors (8 females and 7 males, aged from 25 to 35 years) were selected in terms of meeting the following criteria: no antibiotics therapy and probiotics supplementation at least 6 months before donation, not obese, body weight index (BMI) ≤ 25 kg/m^2^, no chronic and gastrointestinal diseases, no smoking, and being on a traditional European diet (non-excluding meat) (Table S1). Donors collected fresh stool samples at home using a plastic stool collection container (Fisher Scientific, 02-544-208) and were asked to immediately put the container into anaerobic conditions (Genbag anaerobic, BioMerieux) and home refrigerator. The stool samples were transported chilled in an insulated container with an ice pack and processed by the laboratory after a maximum of 8 h from the collection. The stool samples were transferred to an anaerobic chamber (BACTRON 300 Sheldon Manufacturing, Inc., 90% N_2_, 5% H_2_, 5% CO_2_) and portioned into ∼ 3 g samples from each volunteer and pooled from all donor’s stool, next diluted in a cryopreservation buffer (1:4 w/v) (Table S2)^36^, filtered by stomacher bag, aliquoted and frozen at − 80 °C until used for further analysis.

#### In vitro batch cultures of GM samples

Prior to cultures, the frozen pooled faecal (MIX) samples were thawed anaerobically into a water bath (37 °C for 1 h). Next, pre-warmed media: GMM, FM, CFBM, and SM were inoculated with previously frozen faecal microbiota (2% v/v). Batch cultures (n = 6, for each group) were carried out anaerobically in the BACTRON chamber. Samples were incubated on a laboratory shaker (150 rpm) for 24 h at 37 °C using 96 deep-well plates.

#### PMA treatment

Immediately after incubation, samples were treated with propidium monoazide (PMA) (Biotium, Fremont, CA, USA) to discriminate DNA from viable and non-viable organisms. 20 mM PMA™ was added to each sample to a final concentration of 50 µM. Samples were vortexed for 20 s and incubated in the dark at 37 °C for 20 min with vortexing every 5 min. Next, samples were exposed to light using a PMA-Lite LED Photolysis Device (Biotium, Fremont, CA, USA) for 20 min with vortexing every 5 min. Cells were pelleted by centrifuging at 5000×*g* for 10 min, and the supernatant was discarded. For the non-PMA-treated aliquots, samples were kept at room temperature for the duration of the PMA treatment of the paired aliquots. Pellet was stored at − 80 °C until DNA isolation. DNA was extracted from the cell pellets alongside the PMA-treated aliquots.

#### DNA extraction, 16S rDNA amplicon library preparation and MiSeq sequencing

Total DNA from faecal samples was isolated using QIAamp PowerFecal Pro DNA Kit (Qiagen, Hilden, Germany) according to the manufacturer’s instructions. To ensure complete homogenization of the faecal samples, cells were mechanically disrupted by bead-beating using a FastPrep-24 Classic Grinder (MP Biomedicals, Santa Ana, California, USA). Bead-beating was conducted three times for 20 s at 6.5 m/s. The purity of the extracted DNA was analysed using an ND-1000 spectrophotometer (NanoDrop Technologies, Wilmington, DE) and DNA concentration was quantified by a Qubit 4.0 Fluorometer (Life Technologies Ltd., UK) using the Qubit dsDNA BR Assay Kit (Invitrogen). The primer pairs 341 (F: 5′-CCTACGGGNGGCWGCAG-3′) and 805 (R: 5′-GACTACHVGGGTATCTAATCC-3′) including Illumina MiSeq sequencing adapters (Illumina guide protocol #15044223 Rev. B) were used to amplify the V3-V4 region of the 16S rDNA gene. The DNA library preparation was performed according to Juszczuk-Kubiak et al.^37^. Briefly, for each sample, 5 ng of bacterial DNA was amplified in a total reaction of 25 µl with 12.5 µl of 2× KAPA HiFi HotStart ReadyMix (Kapa Biosystems, Roche, Pleasanton, CA, USA) and 1 µM of forward and reverse primers (10 µM). Following PCR conditions were used for amplifications: initial denaturation was at 95 °C for 3 min, and 25 cycles of 95 °C for 30 s, 55 °C for 30 s, 72 °C for 30 s, with the final extension of 72 °C for 5 min. Following PCR amplification PCR products were cleaned using MagSi-NGS^PREP^PLUS (Steinbrenner Laborsystemeand, Germany) and quantified using the Qubit DNA BR Assay Kit (Invitrogen) in conjunction with a Qubit 4.0 Fluorometer (Life Technologies Ltd., UK). To verify the correct amplicon size (~ 550 bp) the TapeStation 4200 platform (Agilent Technologies, Santa Clara, CA) with Agilent D1000 ScreenType Assay Kit was used. 16S rDNA amplicons were indexed using the Nextera XT kit (Illumina, San Diego, CA, USA) according to the Nextera DNA Sample Preparation Guide (protocol #15044223 Rev. B). Each index PCR reaction contained 5 μl of the i7 and i5 adapter, 25 μl of KAPA HiFi HotStart ReadyMix, 5 μl of template DNA, and 10 μl of PCR grade H_2_O for a total reaction volume of 50 μl. The indexed PCR was cycled according to the Nextera DNA Sample Prep Guide, and the libraries were cleaned up using MagSi-NGS^PREP^PLUS (Steinbrenner Laborsysteme, Germany). The DNA libraries were quantified using the Qubit 4.0 along with the Qubit DNA HS Assay Kit, and the quality was assessed on a TapeStation 4200 platform using the High Sensitivity D1000 SreenTape Assay Kit (Agilent, Technologies, Santa Clara, CA). Indexed libraries were normalized to 4 nM and pooled. The normalized, pooled 4 nM library was denatured using 0.2 N NaOH and diluted to 10 pM using prechilled HT1 buffer supplied in the Nextera XT Kit (Illumina, San Diego, CA, USA). The 10% of denatured PhiX library (Illumina, San Diego, CA, USA) was spiked into the denatured and indexed library, which was loaded into Illumina MiSeq v3 reagent cartridge, and 16S rDNA gene amplicons were sequenced on an Illumina MiSeq platform using the 600 cycles (2 × 300 bp) v3 chemistry.

#### Microbiota community analysis

Raw 16S rDNA sequence reads were demultiplexed into FASTQ files, subjected to quality control (> Q30 quality score) and denoised using DADA2 v2022.8.0^38^. Quality control, visualization, taxonomic profiling, and *β*-diversity analysis were conducted using open-source software QIIME 2 v2022.8.3 (https://github.com/qiime2/qiime2)^39^. The table of amplicon sequence variants (ASV) was filtered and only those present in a relative abundance of ≥ 0.25% of the total reads in at least one sample according to the threshold proposed by Reitmeier et al.^40^. Core microbiomes at 100% were computed in QIIME 2 and Venn charts were obtained using the online tool available at http://www.interactivenn.net/^41^. Taxonomic classification was performed using the SILVA database v132 (https://www.arb-silva.de/). PICRUSt2 (Phylogenetic Investigation of Communities by Reconstruction of Unobserved States) v2.5.0 (https://github.com/picrust/picrust2) predictions of metagenome functions^42^ was based on the Kyoto Encyclopedia of Genes and Genomes (KEGG)^43^. Next, QIIME2 artifacts were imported into R v3.6.3 as a phyloseq object using qiime2R v0.99.20 (https://github.com/jbisanz/qiime2R). α-diversity analysis was performed using the phyloseq v1.30-4 (https://github.com/joey711/phyloseq) and vegan v2.6-4 (https://github.com/vegandevs/vegan) R packages to calculate Richness and Shannon effective counts with the rarefaction set to minimum sum count (40,192). Nonmetric Multidimensional Scaling (NMDS) plot based on *β*-diversity distances was obtained using the MicrobiomeAnalyst 2.0 web tool (https://www.microbiomeanalyst.ca/)^44,45^. Taxonomic and functional profiles were further analyzed in STAMP software v2.1.3^46^. Linear discriminant analysis coupled with effect size (LEfSe) v1.1.2 was conducted to identify bacterial taxa differentially represented between different groups at genus or higher taxonomy levels^47^.

#### Determination of microbial SCFAs profile

The 100 µl of the sample was mixed with 500 µl of n-hexane and 500 µl of internal standard (1 µg/ml nonanoic acid solution in isopropanol). After brief mixing, the sample was dehydrated with sodium sulfate. Next, the sample was transferred into a glass vial and capped tightly after adding 10 µl of concentrated sulfuric acid and heated on a thermoblock (1 h, 100 °C) to derivatize. The sample was extracted by adding 4 ml methyl tert-butyl ether and 4 ml of a solution containing sodium citrate and sodium chloride (10 and 15 respectively). After the intensive shaking sample was centrifuged, and 2 ml of the sample from the upper organic phase was moved to a glass vial containing sodium sulfate to remove residual water. The sample was filtered and loaded on GC–MS (Agilent 7890A/5975C, Santa Clara). Separations were performed using RTX-WAX MS capillary column (60 m × 250 µm × 0.25 µm). The injector temperature, ion source, quadrupole, and GC/MS interface were 230, 230, 150, and 250 °C, respectively. The flow rate of helium carrier gas was kept at 1.5 ml/min. 2 µl of the derivatized sample was injected with a split ratio of 50:1. The initial column temperature was 40 °C and held for 2 min, ramped to 60 °C at the rate of 5 °C/min, and then increased to 150 °C at the rate of 8 °C/min and then finally increased to 250 °C at the rate of 15 °C/min and kept at this temperature for 5 min. The ionization was carried out in the electron impact (EI) mode at 70 eV. The MS data were acquired in full scan/SIM mode. The identification of compounds was confirmed by injection of pure standards and a comparison of the retention time and corresponding MS spectra. Standard calibration curves were obtained using authentic analytical standards of SCFAs.

#### SCFAs recovery assay

Post-culture faecal samples were spiked with two different levels (1000 µg/ml, 2000 µg/ml) of mixed SCFA working standards (2 mg/ml). The SCFAs were extracted, derivatized, and analyzed by GC/MS as described above. The spiked recoveries were calculated using the following equation:

$$Recovery\%= \frac{Final \, concentration-Initial \, concentration}{Spiked \, concentration} \times 100 \%.$$

#### SCFA limit of detection and limit of quantification

The limit of detection (LOD), and the limit of quantification (LOQ) were determined based on the standard deviation (SD) of the y-intercept of the regression line (s) and the slope of the calibration curve (S) using the following equations:

$$\mathrm{LOD }= 3.3 \times \left(\frac{s}{S}\right),$$

$$\mathrm{LOQ }= 10 \times \left(\frac{\mathrm{s}}{\mathrm{S}}\right).$$

#### Untargeted metabolomics by LC-HR-MS/MS

The post-culture fluids were mixed with acetonitrile (50:50) and filtered using a nylon syringe filter (0.22 µm) into a chromatographic vial, loaded on LC-HR-MS/MS using a Q Exactive Orbitrap Mass Spectrometer (Thermo Scientific). Metabolites were injected on a C18 column (ACQUITY UPLC HSS T3 1.8 µm; 2.1 × 100 mm). LC was run at a constant flow rate of 0.3 ml/min in a mobile phase gradient composed of A (1 mM ammonium fluoride in water: methanol, 90:10 v/v) and B (1 mM ammonium fluoride in water: methanol 20:80 v/v). All samples were acquired in positive and negative ionization mode. The mass spectrometer was operated at a resolution of 70,000 in simultaneous scan/all ion fragmentation mode. Ionization the parameters were set up as follows: spray voltage (+) 3500 V; spray voltage (−) 2500 V; probe heater temp. 412 °C; capillary temperature 256.25 °C; and gas flows: probe heater temp. 412 °C; sheath gas 47.50; aux gas 11.25; spare gas 2.25. Xcalibure v4.2.47 (Thermo Fisher Scientific, Austin, TX, USA) software was used for data acquisition. Mass tolerance was set at 5 ppm and minimum peak intensity at 100,000. Compound Discoverer v3.2 software was used for peak identification, integration of mass spectrometric data and untargeted metabolomic analysis. The compounds were identified and grouped based on the ChemSpider (https://www.chemspider.com (accessed on 17 February 2022)), mzCloud (https://www.mzcloud.org/ (accessed on 17 February 2022)), and KEGG Pathways (https://www.genome.jp/kegg/compound/ (accessed on 17 February 2022)) databases.

### Statistical analysis

All statistical tests were performed using Graph Prism v9.4.1 (GraphPad Software Inc., San Diego, CA) unless otherwise stated. Differences in *α*-diversity comparisons were assessed using a Mann–Whitney test and Kruskal–Wallis test with the Dunn *post-hoc* test for multiple groups comparisons. Differences in *β*-diversity were evaluated by permutational multivariate analysis of variance (PERMANOVA) performed on Bray–Curtis distances within QIIME2 software. Differences in the relative abundance of GM taxa were made by using Kruskal Wallis H-test with the Games-Howell *post-hoc* test using STAMP software. Differences in PICRUSt2 predicted GM metabolic functions were evaluated by White’s non-parametric *t*-test corrected with Benjamini–Hochberg FDR using STAMP software. Linear discriminant analysis (LDA) effect size (LEfSe) was calculated using the online Galaxy web application provided through the Huttenhower lab (https://galaxyproject.org/learn/visualization/custom/lefse/)^47^. First, differential abundant features were detected using Kruskal–Wallis sum rank test (*α* = 0.05). Next, the pairwise Wilcoxon rank-sum tests (*α* = 0.05) were used to assess the phylogenetic consistency. All-vs-all classes were compared and a linear discriminant analysis score value of 2.0 was chosen as the threshold for discriminatory features. Differences in SCFAs content between groups were analyzed using the ordinary one-way ANOVA followed by Tukey’s test. Statistical evaluation of the metabolomics data was performed using Compound Discoverer software. Hypothesis test was performed by a one-way ANOVA model with Tukey’s post hoc test with Benjamini–Hochberg correction. Differences between the groups were considered significant at *p* < 0.05, and the data were presented as means ± SD.

## Results

### Effect of pooling individual donor’s samples on GM community composition

**Figure 1:**
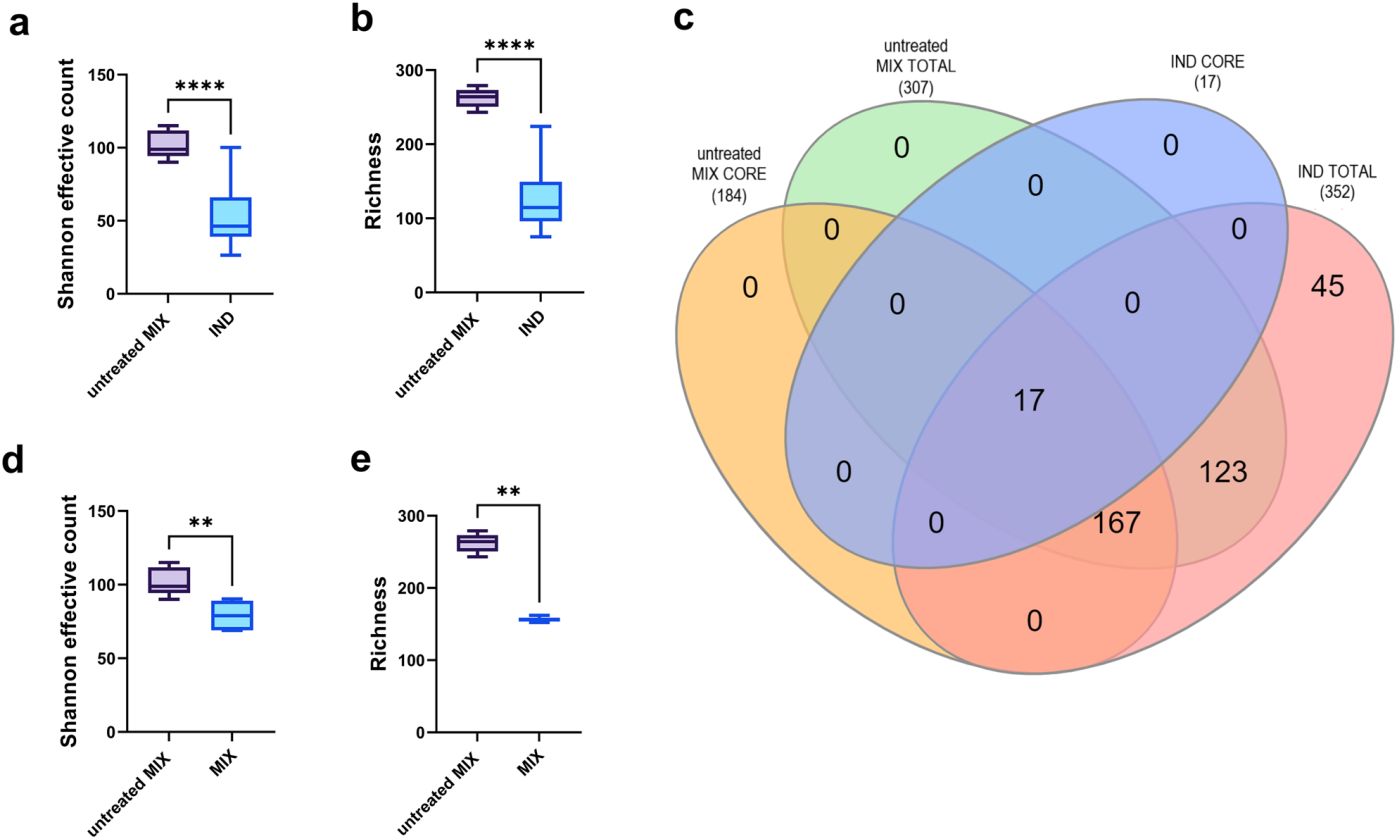
Community *α*-diversity is shown as (**a**) Shannon effective counts and (**b**) Richness between untreated MIX (n = 5) and IND (n = 15) groups. Core microbiota analysis is shown as (**c**) a Venn diagram depicting unique and shared ASVs between MIX (n = 5) and IND (n = 15) samples presented as a number of observed ASVs. Comparison of the MIX samples before (untreated MIX, n = 5) and after PMA treatment (MIX, n = 6) is shown as *α*-diversity by (**d**) Shannon effective count and (**e**) Richness.

First, individual stool samples from 15 healthy donors (IND, n = 15) and pooled stool samples (untreated MIX, n = 5) from all donors without PMA treatment were analyzed by 16S rDNA amplicon sequencing to reveal the impact of sample pooling on microbial community structure (Fig. 1). The comparison of the untreated MIX and IND groups revealed significant differences in *α*-diversity metrics including Shannon effective counts (*p* < 0.0001) (Fig. 1a) and Richness (*p* < 0.0001) (Fig. 1b). Results showed that inoculum from untreated MIX samples was characterized by both the highest species richness and evenness. Next, the specific effect of pooling on the retrieval of total amplicon sequence variants (ASVs) and core microbiome components was assessed for the untreated MIX and IND groups. The 100% core microbiome (ASVs present in all samples) was plotted against the total number of the ASVs for both IND and untreated MIX samples to show the number of unique members of the GM population as well as a representation of the core microbiome found in each group (Fig. 1c). Results showed that in the IND group, the total number of unique ASVs was 352, and only 17 ASVs were present in all donor samples constituting its core. In untreated MIX, the total number of unique ASVs was 307 whereas 59.93% (184) ASVs were present in all the untreated MIX samples (MIX core). Regarding the IND group, 12.78% of all ASVs found in the IND group were not represented in the untreated MIX group. Core ASVs present both in all IND and untreated MIX samples constituted 2.58% of all observed ASVs and were assigned to 6 genera, including *Faecalibacterium*, *Blautia*, *Eubacterium*, *Dorea*, *Bacteroides*, and *Anaerostipes*. Differences in relative abundances of the GM between IND and untreated MIX samples at the phylum level are shown in the table (Table S3). The most abundant phylum both in the IND and untreated MIX samples were *Firmicutes* (65.74% ± 11.96; 70.68% ± 5.21) followed by *Bacteroidetes* (19.88% ± 11.36; 16.43% ± 3.15), *Actinobacteria* (12.57% ± 10.85; 10.99% ± 1.91) and *Proteobacteria* (1.12% ± 1.04; 1.23% ± 0.47). *Verrucomicrobia*, *Tenericutes*, *Euryarchaeota*, and *Cyanobacteria* in total accounted for less than 1% of relative abundance in IND and untreated MIX group. No significant differences in the relative abundance of these taxa between IND and untreated MIX groups were noticed.

To estimate the contribution of the viable bacteria, MIX samples were treated with a PMA viability dye. The comparison of the MIX samples before (untreated MIX) and after PMA treatment (MIX) showed a significant decrease in* α*-diversity metrics including Shannon effective counts (*p* < 0.01) (Fig. 1d) and Richness (*p* < 0.01) (Fig. 1e).

### Comparison of media composition

In this study, the impact of four types of culture media such as GMM, SM, FM, and CFBM on human GM cultivation was investigated (Table S4). GMM was the only medium containing added carbohydrates as an energy source. In turn, SM was the richest in proteins, peptides, and amino acids, and contained over 3 times more nutrients than GMM. In contrast, FM was characterized by the lowest nutrient concentration which contained less than 2 g/l of nutrients. From all tested media, FM and GMM contained the greatest variety of added micro- and macro elements and vitamins and were the only media tested to which various SCFAs were added. In addition, the GMM contained over 14 times more SCFAs per litre than the FM. All tested media contained l-cysteine at the same concentration as a reducing component, while the SM’s additional reducing component was sodium thioglycolate. In each case, resazurin was used as an indicator of the oxygenation of the medium (GMM 1 mg/l; SM 1 mg/l; FM 1 µg/l; CFBM 1 g/l). Only one medium (FM), contained an organic buffering component (HEPES). Additional substances present in the media were hemin, hematin, sheep blood, bile salts, thiacetic acid, and Tween 80. The pH of the investigated microbiological media ranged from 6.8 to 7.2 ± 0.2.

### Effect of various cultivation media on GM community

The tested media were inoculated with pooled stool samples (MIX) and incubated in an in vitro batch model for 24 h. Then, post-culture samples were treated with PMA viability dye to remove non-viable bacteria DNA and sequenced using the MiSeq platform to evaluate the impact of each medium on the GM composition. Obtained outcomes were compared to the non-cultured MIX inoculum treated with PMA, representing the viable part of the GM inoculum.

**Figure 2:**
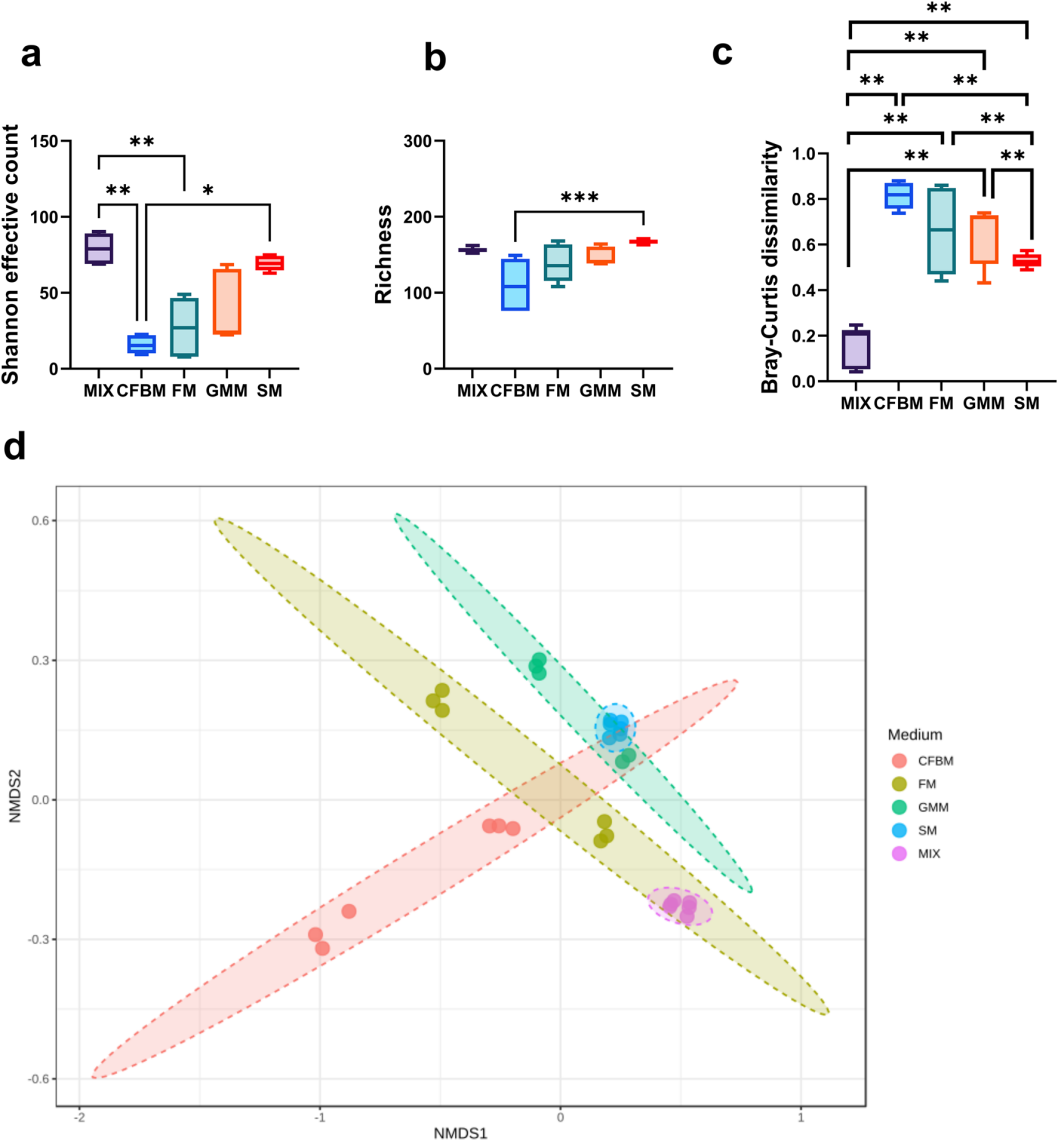
Community *α*-diversity is shown as (**a**) Shannon effective counts and (**b**) Richness of the GM after 24 h of cultivation in four different media: CFBM, FM, GMM, and SM compared to the non-cultured MIX inoculum (n = 6). The *β*-diversity analysis is shown as (**c**) Box plots showing Bray–Curtis distances between microbial communities. (**d**) NMDS plot based on the Bray–Curtis distances metric (R^2^: 0.70263; *p* = 0.001; NMDS stress = 0.0548). Ellipses were drawn based on the 95% confidence interval for each group. Samples represent viable bacteria after PMA treatment.

Using two *α*-diversity metrics, the Richness and Shannon effective counts effect of four types of culture media was analyzed (Fig. 2). After 24 h of cultivation, a significant decrease in Shannon effective counts was noticed between non-cultured MIX inoculum and CFBM, and FM (*p* > 0.01); no significant differences in Shannon effective counts for GMM and SM were found (Fig. 2a). In turn, no significant differences in Richness between non-cultured MIX inoculum and all media groups were found, but a significant reduction of this metric was noticed between SM and CFBM (*p* > 0.001) (Fig. 2b).

PERMANOVA tests performed using Bray–Curtis *β*-diversity metrics showed significant differences in community structure between all media groups and non-cultured MIX inoculum (*p* < 0.001). No significant differences were found between the FM and CFBM (Fig. 2c). SM medium was characterized by the greatest internal homogeneity compared to other media groups (Fig. 2c,d).

**Figure 3:**
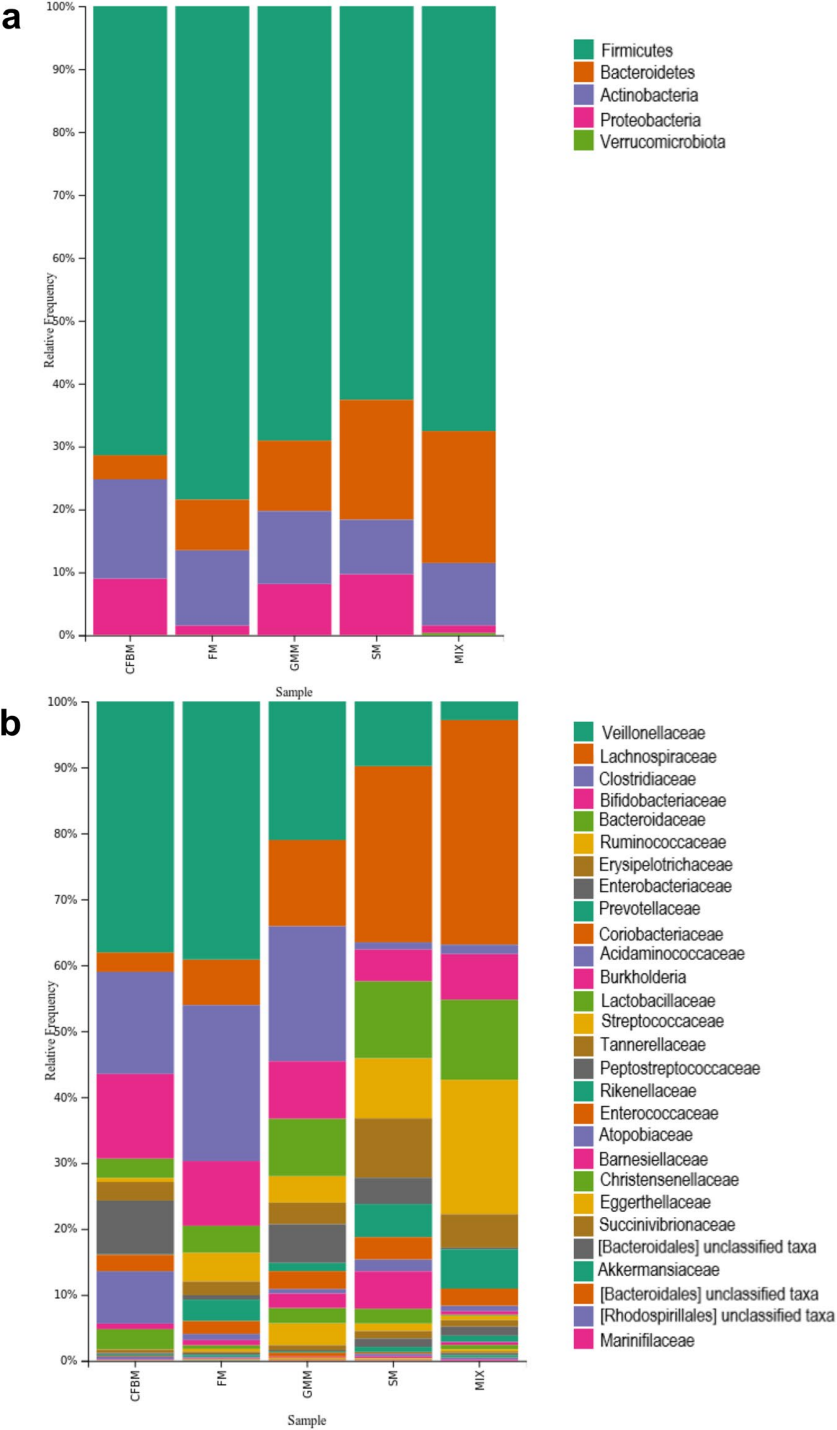
Taxonomic composition of GM communities is presented as the mean of 6 replicates at phylum (**a**) and family (**b**) level after 24 h of cultivation in four different media: CFBM, FM, GMM, and SM compared to the non-cultured MIX inoculum (n = 6). Samples represent viable bacteria after PMA treatment.

The use of various culture media was associated with differences in the relative abundance of GM taxa at both the phylum (Fig. 3a) and family (Fig. 3b) levels. In all tested media, the top most abundant phyla were *Firmicutes* (Table S5). The relative abundance of *Firmicutes* in CFBM, FM, GMM and SM was 71.69% ± 3.91, 74.90% ± 10.67, 68.02% ± 8.79 and 63.16% ± 4.57 (*p* > 0.05), respectively. A significant decrease of *Bacteroidetes* was observed in the CFBM (4.01% ± 3.62) compared to non-cultured MIX inoculum (22.10% ± 7.17; *p* < 0.02) and SM (18.43% ± 5.21; *p* < 0.01) (Table S5, Fig. S1). In comparison to non-cultured MIX inoculum, CFBM, and FM promoted the growth of *Actinobacteria*, whereas GMM and SM increased the growth of *Proteobacteria* (Table S5). Moreover, a significant decrease in the relative abundance of *Verrucomicrobia* phylum was noticed in all media groups compared to the uncultured MIX inoculum (Fig. S1). At the family level, the most abundant taxa were *Veillonellaceae*, *Lachnospiraceae*, *Clostridiaceae*, *Bifidobacteriaceae*, *Bacteroidaceae*, *Ruminococcaceae*, *Erysipelotrichaceae*, *Enterobacteriaceae*, *Prevotellaceae*, *Coriobacteriaceae*, and *Acidaminococcaceae* representing about ~ 90% of the microbial community in all tested culture media (Fig. 3b).

Results showed, that in the non-cultured MIX inoculum *Bacteroides* (12.27% ± 4.45), *Blautia* (10.20% ± 3.35), *Faecalibacterium* (8.31% ± 1.89), *Bifidobacterium* (6.84% ± 0.43), *Prevotella* 9 (5.23% ± 1.19), *Subdoligranulum* (4.08% ± 0.42), unclassified taxa from the *Lachnospiraceae* (4.04% ± 0.95) family, *Agathabacter* (3.69% ± 0.81), *Fusicatenibacter* (3.21% ± 0.68), and *Ruminococcus 2* (3.02% ± 0.19) were most abundant genera (Table S6). In all media groups, we noticed a significant decrease in the relative abundance of *Ruminococcus 2*, *Subdoligranulum*, *Agathabacter,* and *Fusicatenibacter* (Fig. S2). *Faecalibacterium* was significantly less abundant in GMM (*p* < 0.01), SM (*p* < 0.05) and CFBM (*p* < 0.001) while no significant difference in its relative abundance was observed in FM compared to non-cultured MIX inoculum. A relative increase in *Bifidobacterium* abundance was observed in CFBM (*p* < 0.05) and its decrease in SM (*p* < 0.001). Moreover, SM significantly promoted the increase in abundance of the unclassified taxa from the *Lachnospiraceae* family (*p* < 0.01) compared to the non-cultured MIX inoculum. Contrarily, the use of the CFBM inhibited the growth of these taxa (*p* < 0.05).

**Figure 4:**
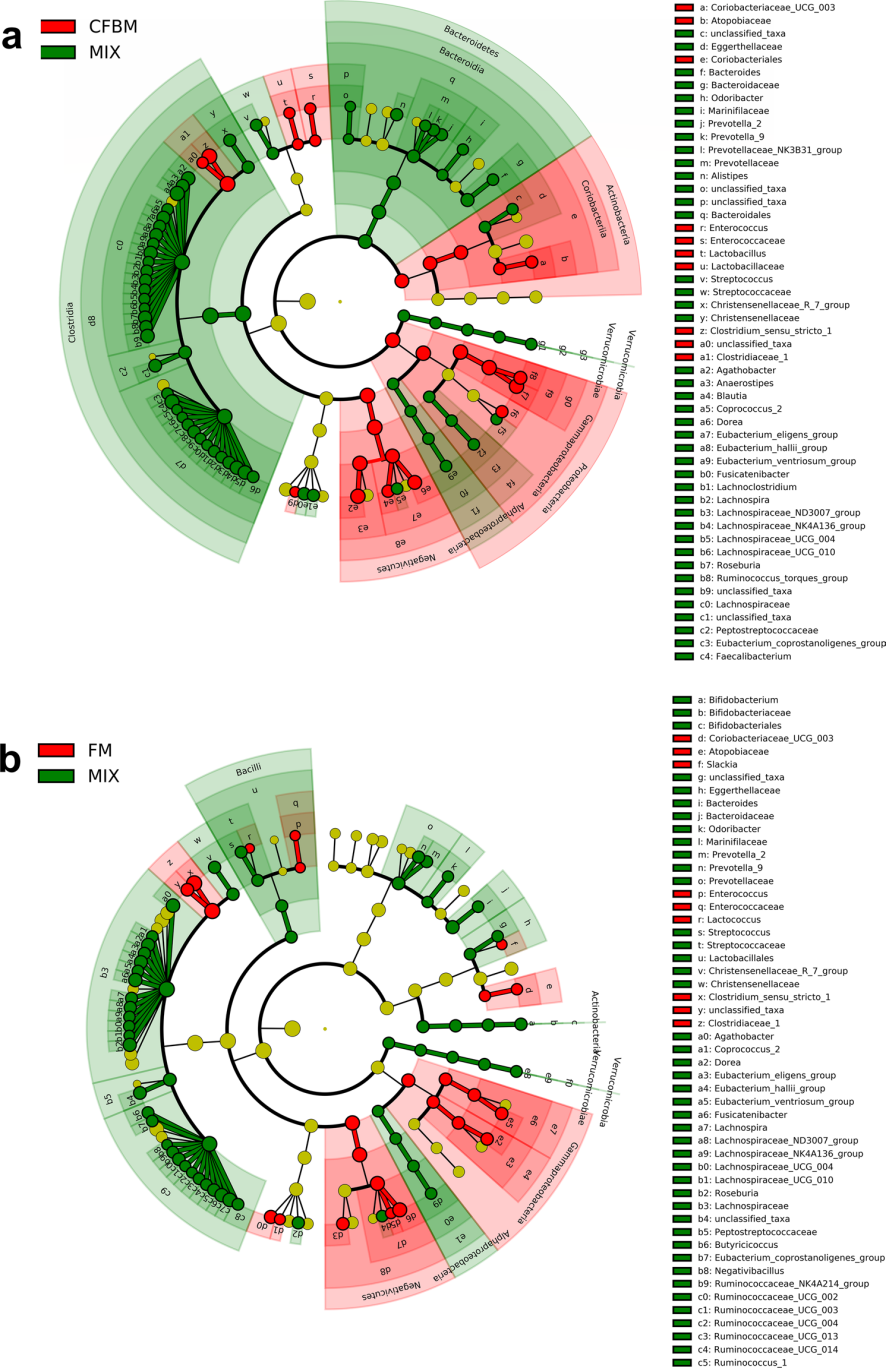

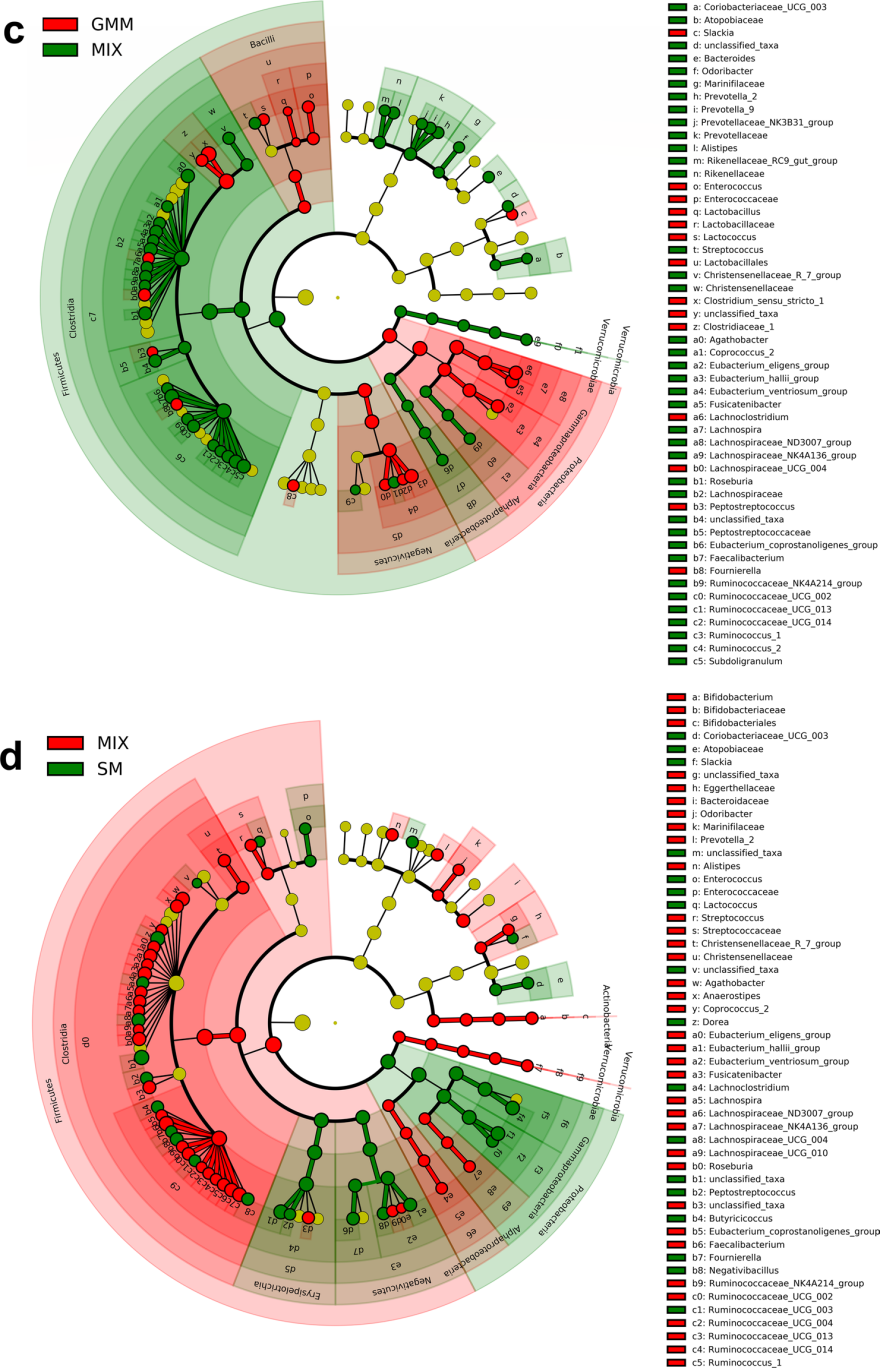
Linear discriminant analysis Effect Size (LEfSe) cladogram of the GM composition after 24 h of cultivation in four different media: CFBM (**a**), FM (**b**), GMM (**c**), and SM (**d**) compared to the non-cultured MIX inoculum (n = 6). Red, green, and nodes/shades indicate taxa that are significantly higher in relative abundance in each group; the diameter of each node is proportional to the taxon’s abundance; each successive circle represents a phylogenetic level. LDA score > 2, *p* < 0.05. Samples represent viable bacteria after PMA treatment.

To identify microbial biomarkers associated with tested media and non-cultured MIX inoculum, linear discriminant analysis (LDA) of effect size (LEfSe) was performed (Fig. S3). Differences between GM communities are represented visually on LEfSe cladograms (Fig. 4). In the CFBM, LEfSe analysis revealed 12 significantly enriched genera, including *Enterobacteriaceae*, *Acidaminococcus*, *Escherichia, Shigella, Megasphera*, and the most dominant *Clostridium sensu stricto 1* with an LDA > 4.8. Whereas 76 phylotypes including 4 classes and 4 orders were more abundant in the non-cultured MIX inoculum compared to the CFBM. At the genus level, the most important representatives were *Lachnospiraceae* and *Ruminococcaceae* with an LDA score > 4.8 (Fig. 4a). In turn, the LEfSe analysis showed 85 differentially abundant bacterial phylotypes between the FM and non-cultured MIX inoculum. The predominance of 13 genera with the most dominant taxa *Clostridium sensu stricto 1* (LDA ≥ 4.8) and underrepresentation of 12 families in the FM compared to the non-cultured MIX inoculum was observed (Fig. 4b). Regarding GMM, an increased abundance of 31 phylotypes and decrease of 57 phylotypes in comparison to the non-cultured MIX inoculum was found. The most prevalent microbial biomarker at the genus level was *Clostridium sensu* *stricto 1* with an LDA score > 4.8 while the most lowered biomarkers belonged to *Firmicutes* phylum and *Clostridia* class (Fig. S3c). Comparison of SM with the non-cultured MIX inoculum resulted in the identification of 99 significantly different phylotypes of which 40 were increased (Fig. S3d). Accordingly, the cladogram shows a predominance of *Gammaproteobacteria*, *Erysipelotrichia*, and *Negativicutes* class (Fig. 4d). At the genus level, unclassified taxa from the *Lachnospiraceae* family, *Catanibacterium*, *Sutterella*, *Dorea Escherichia* and *Shigella* were dominant with an LDA score > 4.2; *Clostridia*, *Actinobacteria*, and *Alphaproteobacteria* class were the most characteristic for the non-cultured MIX inoculum with LDA score > 3.

**Figure 5:**
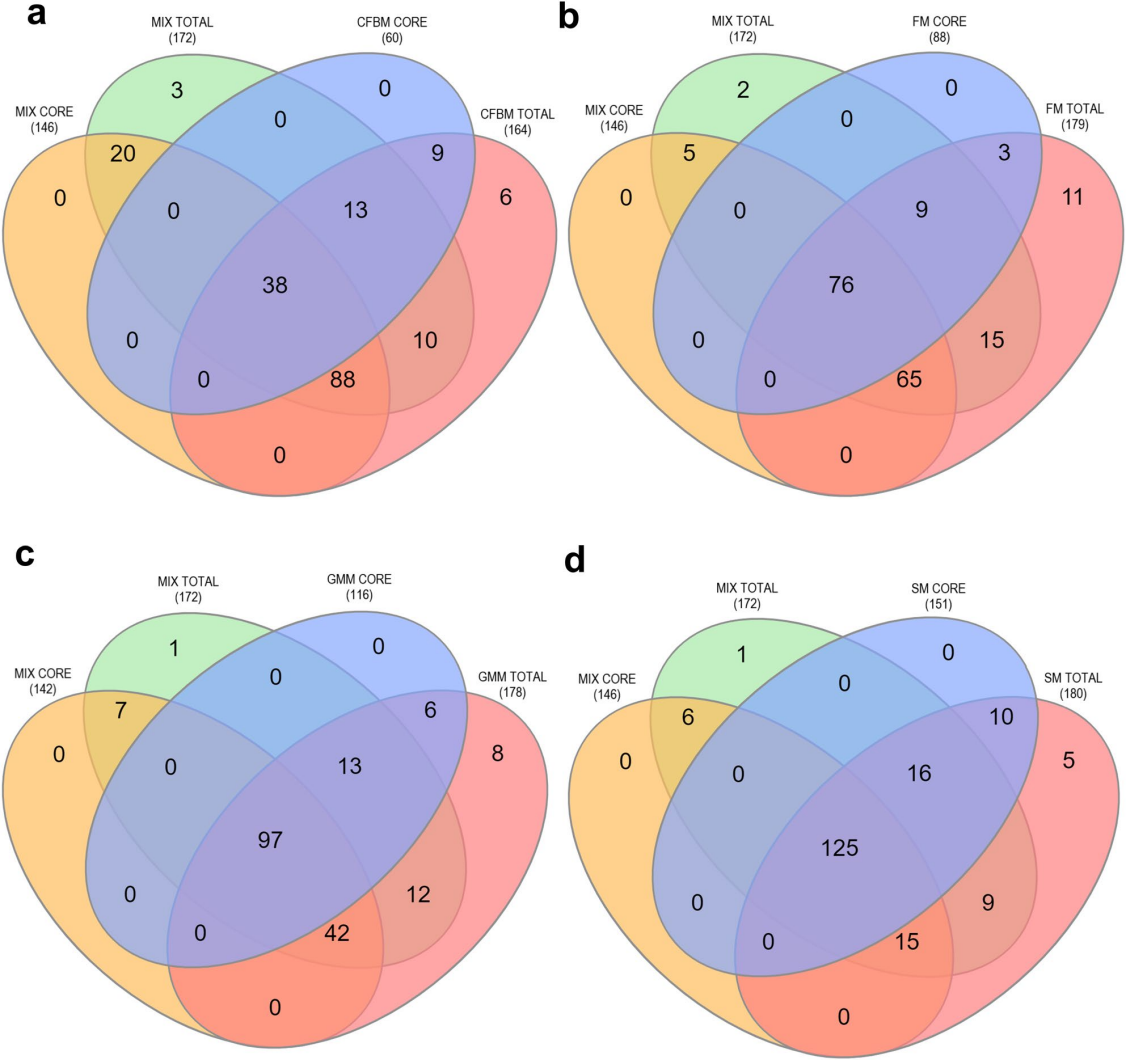
Core microbiota analysis. Venn diagram depicting unique and shared ASVs between CFBM (**a**), FM (**b**), GMM (**c**), and SM (**d**) after 24 h cultivation and non-cultured MIX inoculum is presented as a number of observed ASVs. Samples represent viable bacteria after PMA treatment.

The Venn diagrams show overlap between unique and shared ASVs observed in media groups (CFBM, FM, GMM, SM) after 24 h of cultivation compared to the non-cultured MIX inoculum (Fig. 5). Core microbiota analysis found 38 for CFBM (Fig. 5a), 76 for FM (Fig. 5b), and 97 for GMM (Fig. 5c) shared ASVs with non-cultured MIX inoculum. The highest number of total shared ASVs (125) with non-cultured MIX inoculum was observed in SM medium (Fig. 5d). In total, 149 ASVs for CFBM (90.85%), 165 for FM (92.17%), 163 for GMM (91.57%), and 165 for SM (91.57%) were shared with non-cultured MIX inoculum.

### Functional predictions of the GM communities

**Figure 6:**
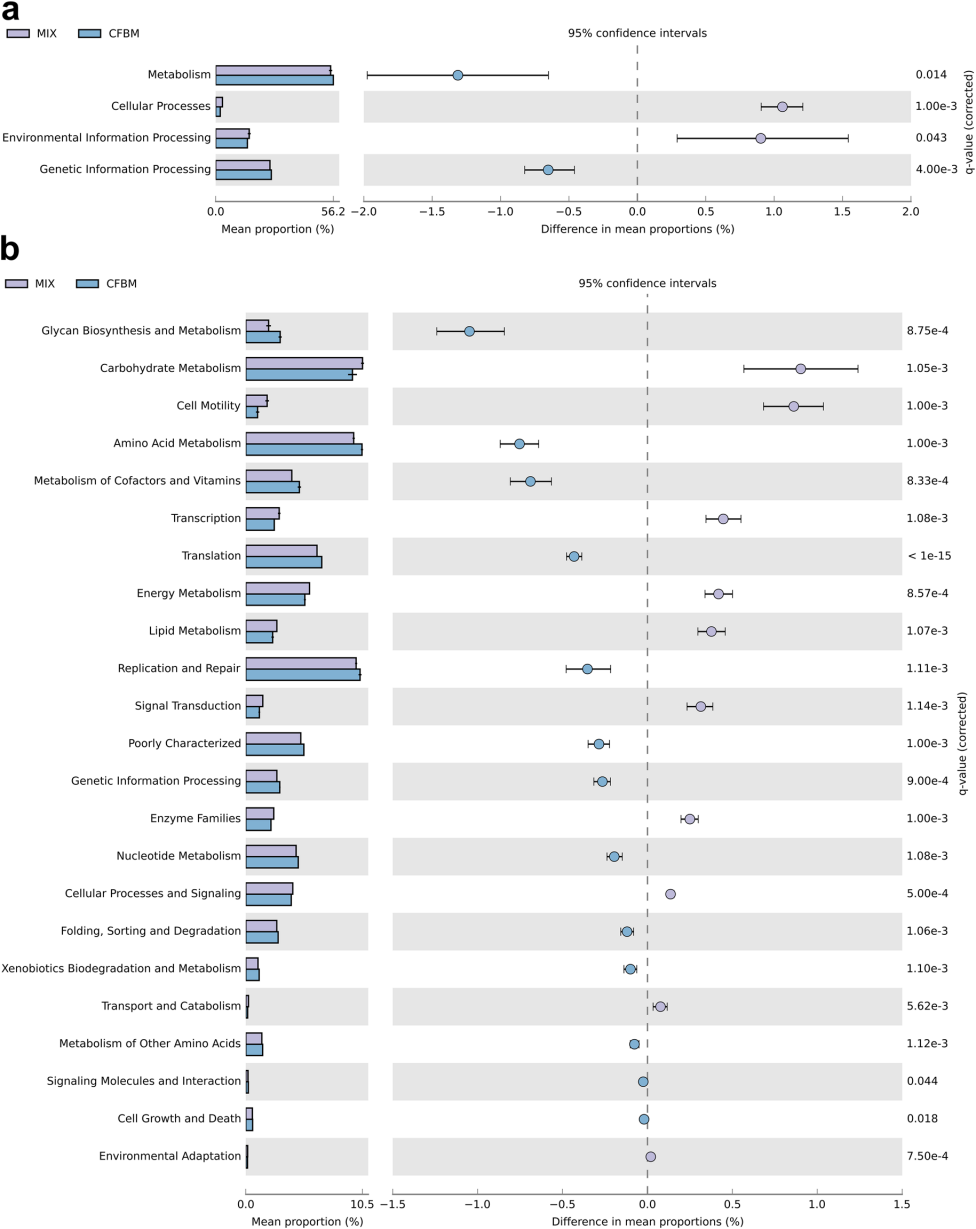

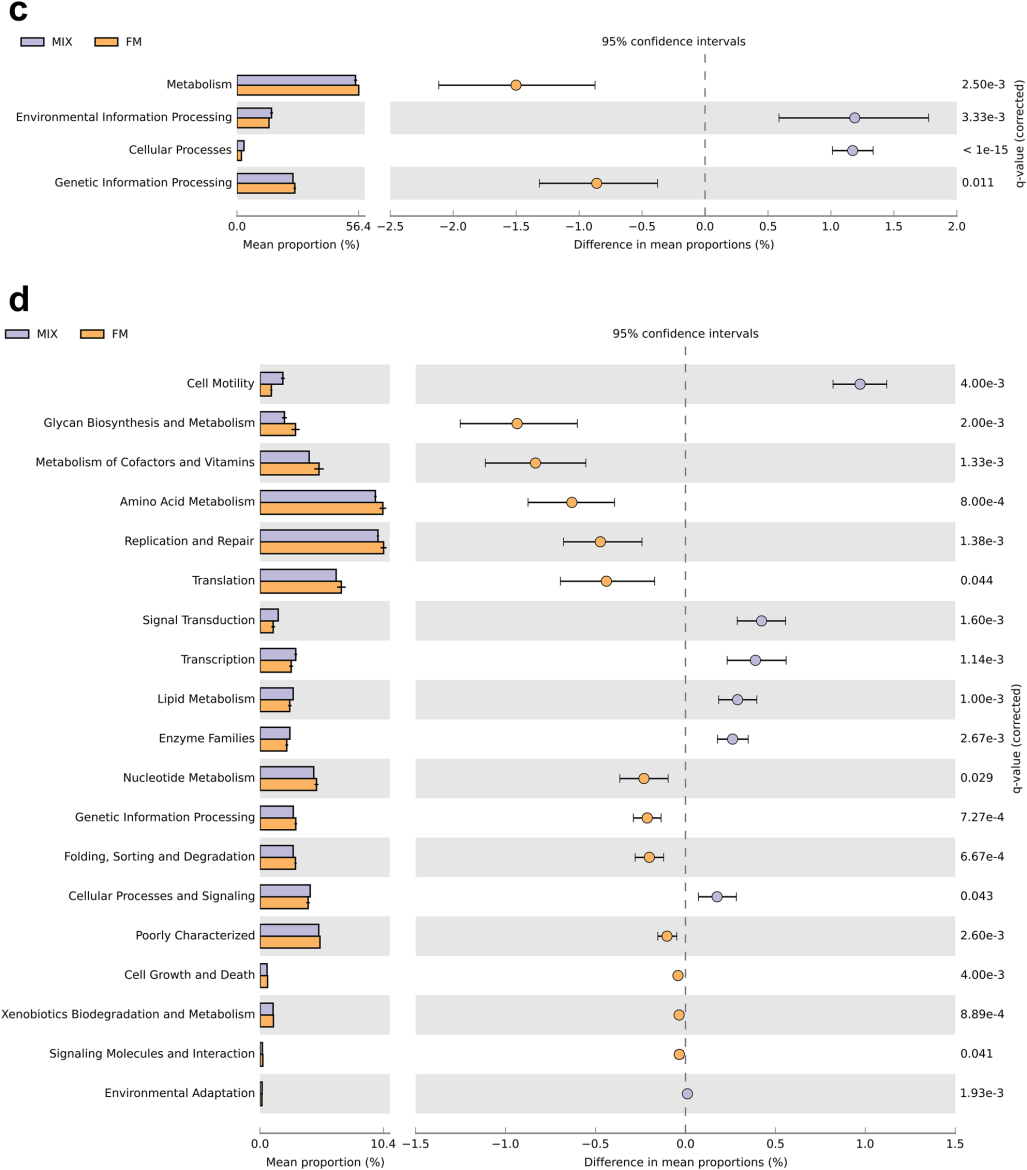

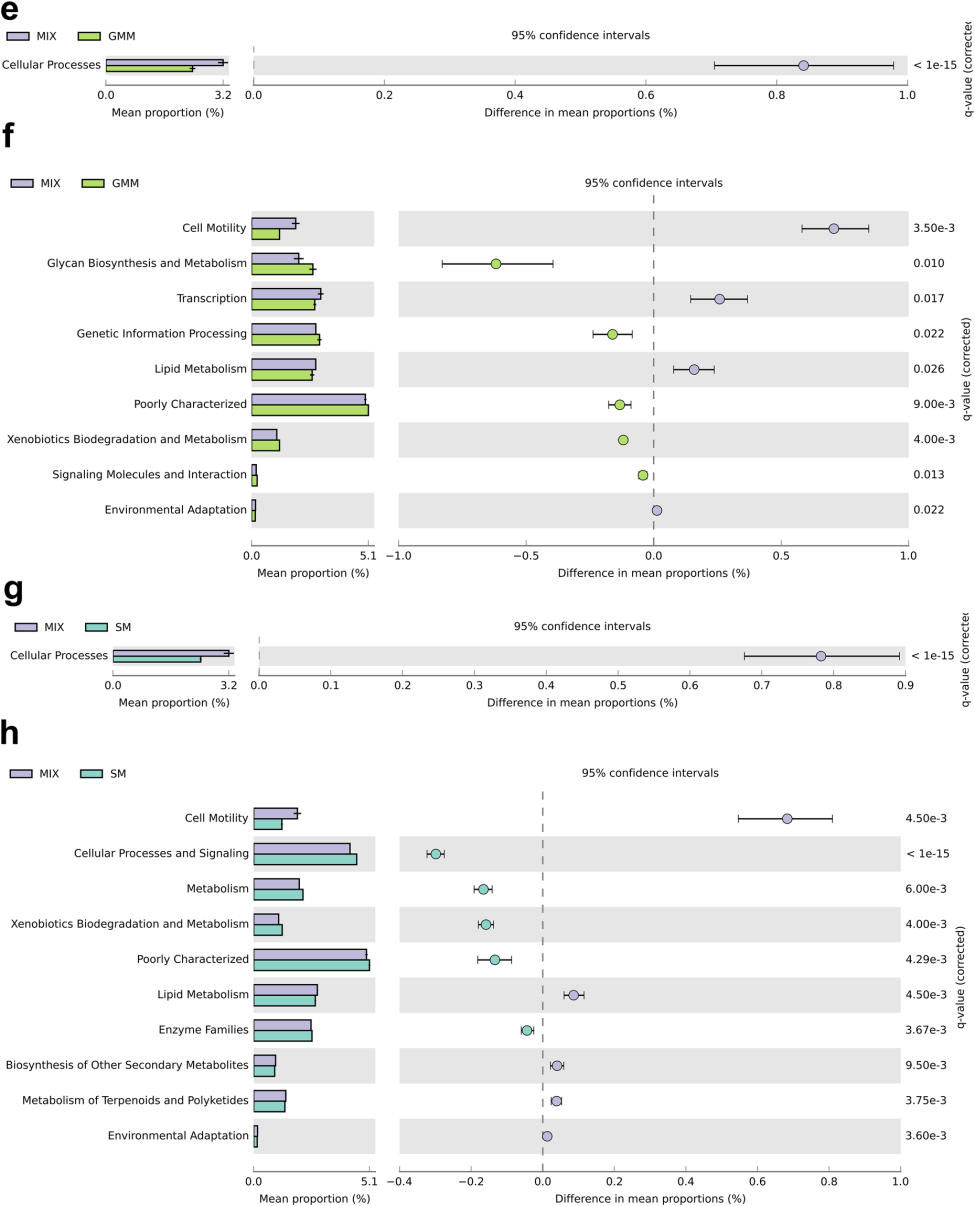
PICRUSt2 predicted GM metabolic functions at KEGG pathway Level 1 (**a**,**c**,**e**,**g**) and Level 2 (**b**,**d**,**f**,**h**). STAMP analysis revealed significantly different metabolic functions between non-cultured MIX inoculum and MIX samples after 24 h of cultivation in four different media: CFBM (**a**,**b**), FM (**c**,**d**), GMM (**e**,**f**), and SM (**g**,**h**). Samples represent viable bacteria after PMA treatment.

PICRUSt analysis based on the 16S rDNA gene sequence data predicted significant differences in the functional potential of the GM between the non-cultured MIX inoculum and CFBM, FM, GMM, and SM groups (Fig. 6, Tables S7–S10).

Finally, KEGG level 1 analysis showed a significant decrease in the relative abundance of cellular processes and environmental information processing pathways in the CFBM compared to the non-cultured MIX inoculum (Fig. 6a). In contrast, metabolism and genetic information processing was significantly increased in the CFBM compared to the non-cultured MIX inoculum. On the KEGG level 2 analysis, 13 of 23 significantly different pathways were elevated, including glycan biosynthesis and metabolism, amino acid metabolism, metabolism of cofactors and vitamins as well as translation. The top 3 most lowered pathways concerned carbohydrate metabolism, cell motility, and transcription (Fig. 6b). The KEGG level 3 analysis showed 159 significantly altered pathways. Among them, the greatest differences were found for the transporters (13.28% decrease), and sporulation (58.00% decrease). The largest increase in abundance was noticed for lipopolysaccharide biosynthesis proteins (69.87%) (Table S7).

In turn, PICRUSt analysis concerning differences between the FM and non-cultured MIX inoculum showed a significant decrease in level 1 KEGG pathway related to environmental information processing and cellular processes whereas metabolism and genetic information processing were significantly increased. However, the most decreased pathway on deeper KEGG level 2 was cell motility (Fig. 6c). The remaining most decreased pathways in the FM were noticed for signal transduction, followed by transcription, lipid metabolism and enzyme families. Most increased pathways compared to the non-cultured MIX inoculum, on KEGG level 2 were related to glycan biosynthesis and metabolism, metabolism of cofactors and vitamins, metabolism of amino acids, replication and repair, and translation (Fig. 6d). Regarding level 3 KEGG, a total of 146 pathways were significantly different between the FM and non-cultured MIX inoculum; the largest difference was encountered for the transporters pathway (12.14% decrease) (Table S8).

At level 1 of KEGG, the cellular processing pathway was significantly decreased between the GMM and non-cultured MIX inoculum (Fig. 6e). On a deeper resolution, including level 2 KEGG, data showed the highest significant decrease of the cell motility, transcription, and lipid metabolism in GMM compared to non-cultured MIX inoculum. In turn, the KEGG pathways related to glycan biosynthesis and metabolism, genetic information processing, and xenobiotic biodegradation and metabolism were significantly enriched in GMM (Fig. 6f). At level 3 KEGG, a total of 84 pathways were differentially abundant between GMM and non-cultured MIX inoculum (Table S9). The highest pathway enrichment in the GMM compared to the non-cultured MIX inoculum was found for the lipopolysaccharide biosynthesis proteins (55.36%), whereas the sporulation pathway was the most significantly lowered among all identified pathways (38.00%).

GM cultivation in the SM medium was associated with a significant (*p* < 0.001) decrease in the level 1 KEGG pathways related to cellular processing (Fig. 6g). Within cellular processing, the level 2 KEGG showed the largest decrease in cell motility in the SM compared to the non-cultured MIX inoculum (Fig. 6h). KEGG pathways associated with cellular processes and signaling, metabolism, xenobiotic biodegradation and metabolism, and enzyme families were more abundant in the SM compared to the non-cultured MIX inoculum, while lipid metabolism, biosynthesis of other secondary metabolites, metabolism of terpenoids and polyketides, and environmental adaptation pathways were significantly lowered in comparison to the non-cultured MIX inoculum. At level 3 KEGG, 99 functional pathways with different abundance between the SM and non-cultured MIX inoculum were identified (Table S10), among which bacterial motility proteins (40.00%), flagellar assembly (58.82%), and bacterial chemotaxis (35.90%) were the most lowered, whereas other ion coupled transporters (14.93%), lipopolysaccharides biosynthesis proteins (39.02%), and lipopolysaccharides biosynthesis (48.39%) were the most elevated.

### Impact of different cultivation media on SCFAs production

The SCFAs calibration curves were generated using least-square linear regression analysis in the range of 125–94,000 µg/ml (Table 1). The calibration equations were obtained from the peak areas of each analyte against the internal standard (nonanoic acid 1 µg/ml). The accuracy and precision were assessed by the average recovery and percentage RSD of three results at each concentration as shown in Table 1. The obtained results were within the acceptance criteria as the average recovery of SCFAs ranged from 84 to 118%, and RSDs ranged from 0.2562 to 1.1381%.

**Table 1:** Analytical parameters of GC/MS analysis of SCFAs.

SCFAs	*t*R (min)	m/z	R^2^	Linearity range (µg/ml)	LOD	LOQ	Rec (%)	RSD (%)
Acetic acid	5.247	87.00	0.9984	125–94,000	9.37	31.24	117	1.1381
Propionic acid	6.203	75.00	0.9978	125–4000	20.68	68.93	118	0.8906
Isobutyric acid	8.121	89.00	0.9985	125–4000	4.12	13.72	102	0.5987
Butyric acid	6.281	89.00	0.9998	125–4000	1.96	6.53	97	0.6954
Valeric acid	11.524	85.00	0.9998	125–4000	0.75	2.50	99	0.2562
Isovaleric acid	9.401	85.00	0.9997	125–4000	3.14	10.48	84	0.5938

*tR* retention time, *m/z* mass to charge ratio, *R*^*2*^ coefficient of determination of linear regression, *LOD* limit of detection, *LOQ* limit of quantification, *Rec* recovery rate, *RSD* relative standard deviation.

**Figure 7:**
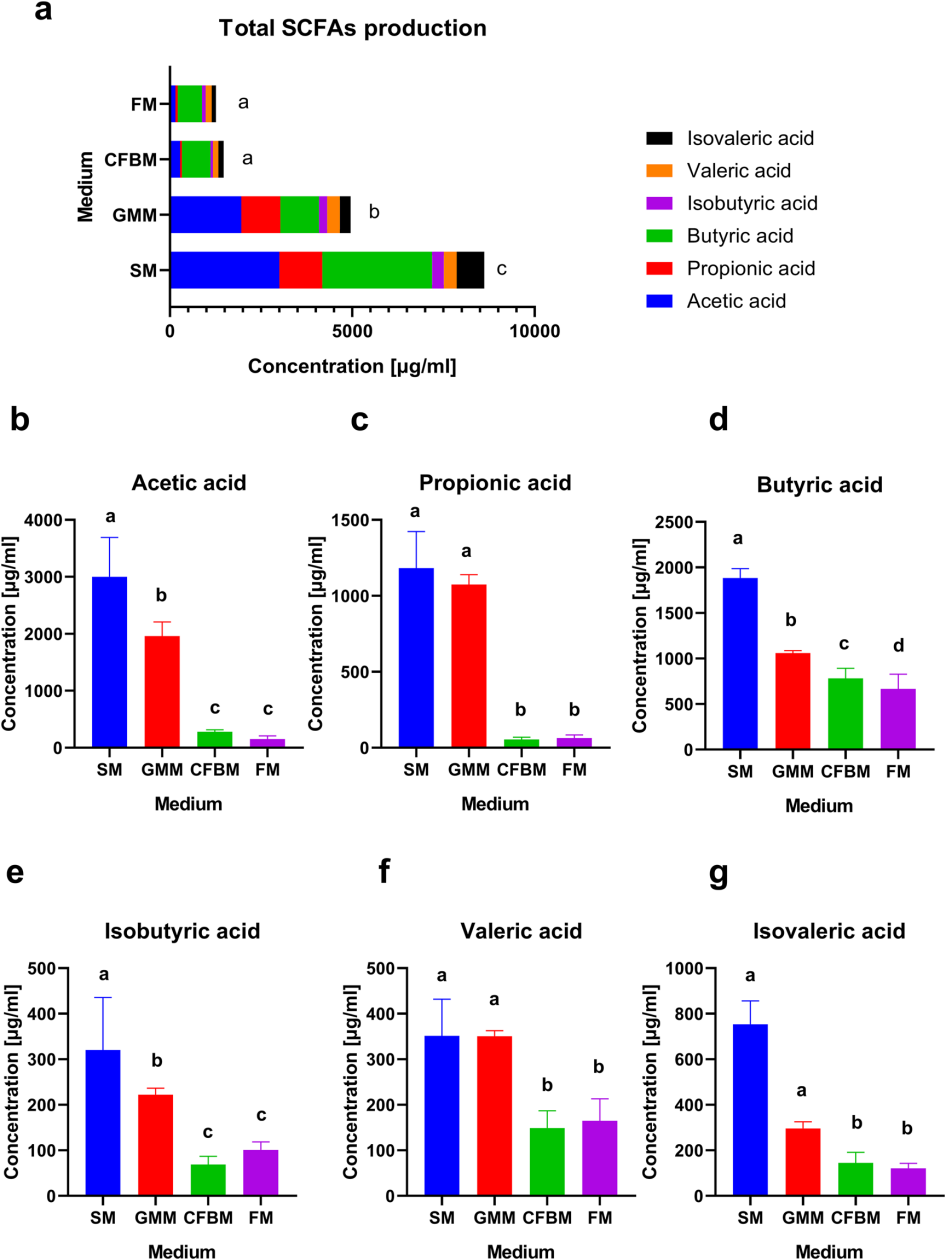

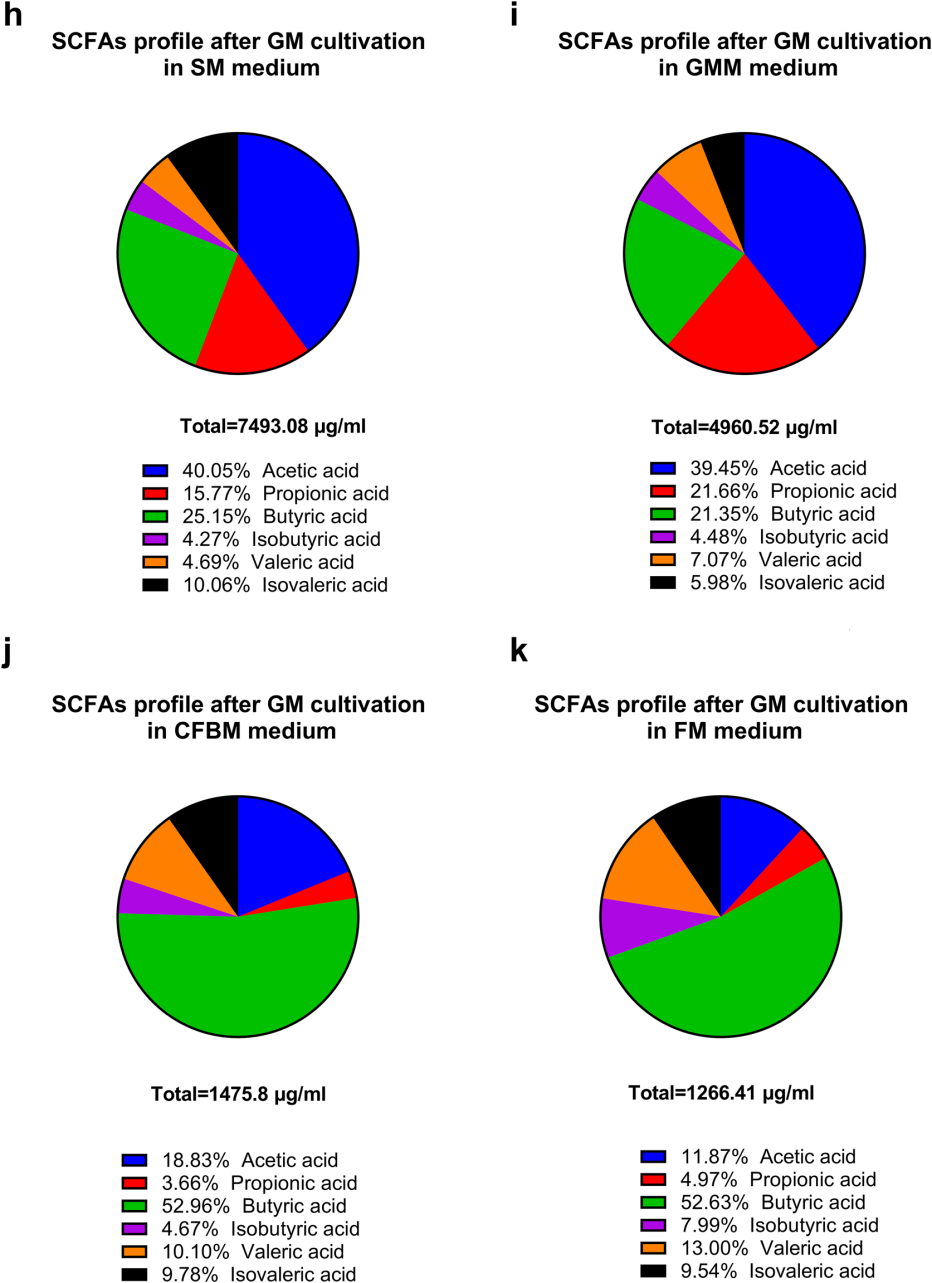
Effect of culture media on the short-chain fatty acids (SCFAs) content after 24 h of cultivation represented as total SCFAs production (**a**), individual SCFAs production (acetic acid (**b**), propionic acid (**c**), butyric acid (**d**), isobutyric acid (**e**), valeric acid (**f**), and isovaleric acid (**g**)) and relative profiles of SCFAs in different media: SM (**h**), GMM (**i**), CFBM (**j**), FM (**k**).

The production of SCFAs, including acetic, propionic, isobutyric, butyric, valeric, and isovaleric acid by GM in four different media was assessed (Fig. 7). The highest total SCFAs production after 24 h culture was observed in the SM with a concentration over 8500 µg/ml, whereas the lowest production was observed in the CFBM and the FM with SCFAs production less than 1500 µg/ml (Fig. 7a). The SM showed the highest production of acetic, butyric and isobutyric acid of all tested media groups (Fig. 7b–g). In turn, the GMM and the SM showed equally high production of propionic, isovaleric, and valeric acid. In both the SM and the GMM groups, acetic acid accounted for the largest proportion of SCFAs produced (40.05% and 38.45%, respectively), whereas the CFBM and the FM were mostly producing butyric acid (52.90% and 52.63%, respectively) indicating large differences in relative SCFAs production profiles (Fig. 7h–k). Moreover, differences in the relative quantity of propionic acid were observed in tested media; it was the third most produced SCFA in the SM (15.77%), second in the GMM (21.66%), sixth in CFBM (3.66%), and FM (4.97%).

### Untargeted metabolomic patterns

**Figure 8:**
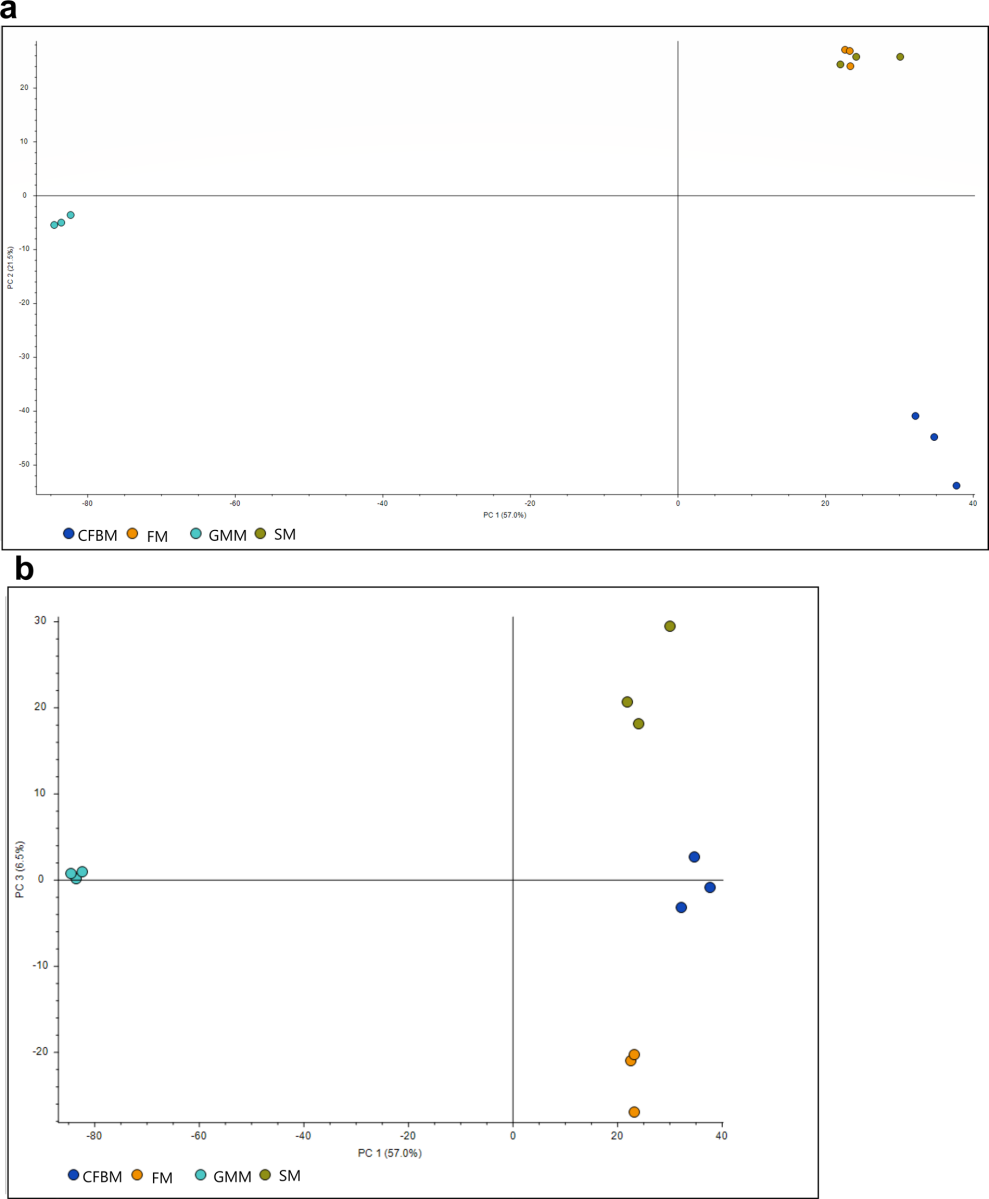
Principal component analysis PC2 *vs* PC1 (**a**), and PC3 for PCA1 (**b**) showing group and separation of metabolite profiles of GM after 24 h of cultivation in different culture media (SM, GMM, FM, CFBM).

**Figure 9:**
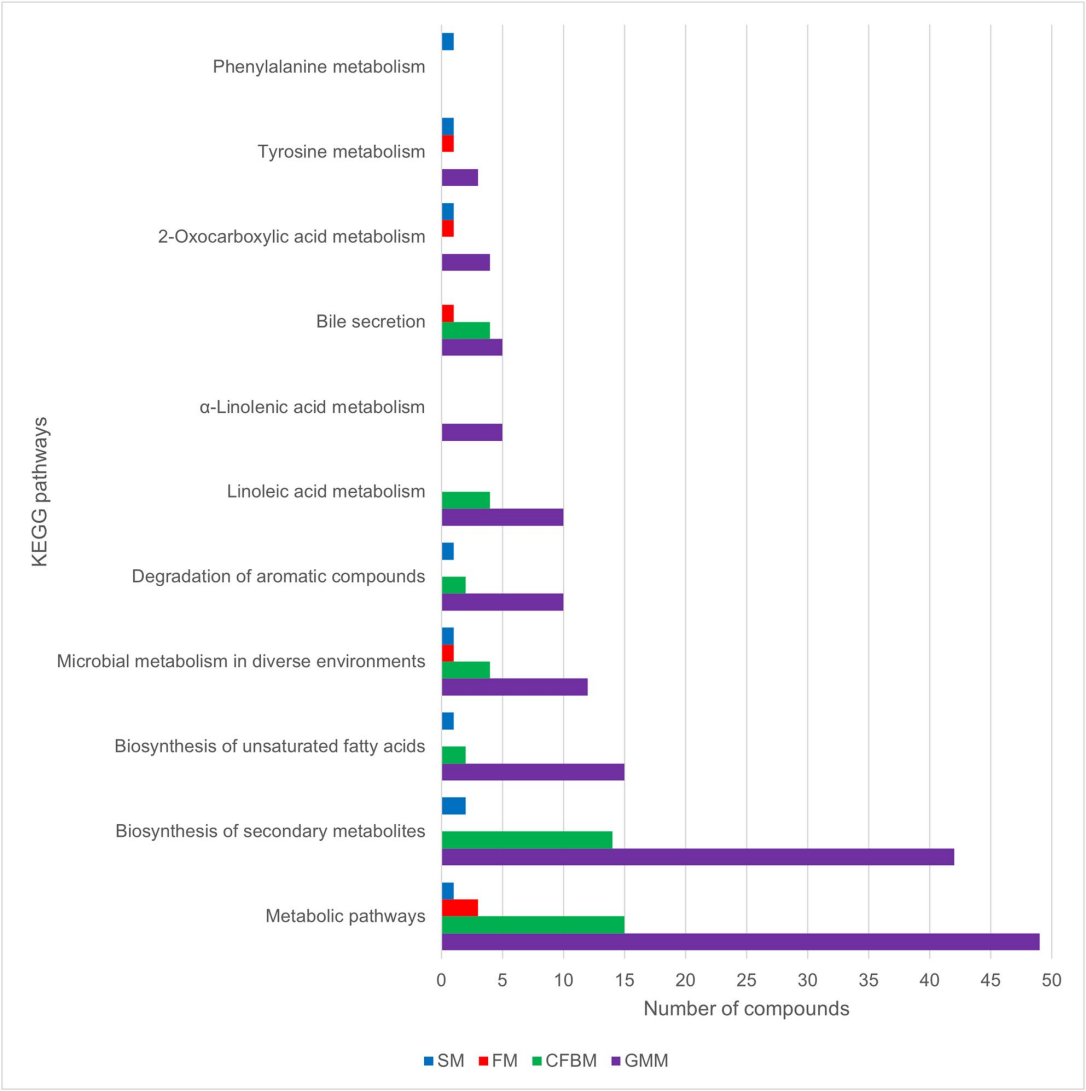
Number of compounds significantly differentiating a given medium (SM, GMM, FM, CFBM) assigned to main metabolic pathways according to the KEGG database.

A total of 37,084 microbial metabolites were detected (peak area > 10,000,000) in faecal samples after cultivation in the GMM, FM, SM, and CFBM using LC-HR-MS/MS approach. In total, 4484 compounds were significantly different (*p* < 0.01) between tested media groups, but 79% of these metabolites were unidentified. These differences were shown in PCA plots which explained 85% of the total variation in the three primary principal axes (Fig. 8). PC2 *vs* PC1 plot (Fig. 8a) showed similarities between SM and FM groups. However, the PC3 *vs* PC1 plot (Fig. 8b) showed a clear separation of samples cultured in all media groups. GM cultivation in GMM resulted in the most distinct metabolome profile compared to other media (SM, FM, and CFBM) according to cluster analysis. In turn, SM and FM shared the most similar metabolome among all tested media groups (Fig. 8b). The filtering for compounds significantly differentiating a given medium (e.g., GMM vs. FM/SM/CFBM *p* < 0.01 (in all three setups)) did also show the increased profile specificity in the case of GMM (2229 of such compounds), in comparison to other tested media (FM—72, SM—95, CFBM—765 compounds). The highlighted compounds were matched to such KEGG pathways as metabolic pathways, bile secretion, biosynthesis of secondary metabolites, biosynthesis of unsaturated fatty acids, and microbial metabolism in diverse environments (Fig. 9).

## Discussion

The gut microbiota (GM) plays a key role in shaping human health and has been the subject of an increasing number of studies in the context of disease development^8–11^. Important progress has been made, especially in the development and optimization of suitable culture media for complex GM in vitro cultivation^14,16^. Nonetheless, to date, a large number of GM species still lack a cultured representative, which can be metabolically active in the human gut environment. Currently, little consensus exists as to the best standard protocols for GM in vitro culture, which could be utilized in various analyses of the human GM.

In this work, we evaluated the impact of four types of culture media to maintain human GM taxon-function stability and the utility of a suitable medium for further toxicomicrobiomics studies. In the first step of the experiment, we determined whether pooled faecal inoculum can be used as an alternative for individual donor faecal for in vitro cultivation in tested culture media. Pooling faecal samples in the present study allowed for the elimination of individual variability as a source of error in the results. Next, the effect of pooling on the retrieval of total ASVs and core microbiome was evaluated. Finally, integrative analysis of faecal 16S rDNA amplicon sequencing and metabolome profiling after 24 h in vitro batch cultivation was performed.

### Effect of faecal sample pooling on GM community composition

The faecal samples collected from individual donors are characterized by a high diversity of microbial community signatures in terms of species composition and abundance pattern^29,48^. To date, despite growing knowledge in microbiome studies, it remains challenging to define the microbial features that universally characterize a healthy human microbiome. Based on research carried out, it is believed that the pooling of faecal samples from different individuals to prepare standardized inoculum is a valuable measure in population-level association research studies^48–50^. The combined faecal inocula are assumed to have a more heterogeneous population and are, therefore, more representative microbiota for the whole population^30^. Moreover, the use of pooled GM as an inoculum for in vitro cultivation models allows using standardized inoculum for multiple different trials and ensures reproducibility between experiments^19,51,52^ The suitability of pooled microbiome samples in cultivation in vitro models has been reported in human adults^19^ and infants’ faecal^31^ as well as human milk^53,54^.

In our study, to minimize the effect of individual variation in the faecal microbiota, an inoculum from pooled faecal samples (MIX) including 15 healthy adult donors has been prepared. Next, we compared the faecal inoculum from individual donors (IND) with the inoculum from mixed samples (MIX) of all donors to investigate the effect of the sample pooling on the GM community composition by *α*-diversity metric, core microbiota analysis, and relative abundance analysis. First, we evaluated the specific effect of pooling on GM *α*-diversity using Richness and Shannon effective count. Richness accounts for the number of taxa above a fixed threshold of relative abundance (0.25%) according to the concept of “Effective microbial richness” (EMR) proposed by Reitmeier et al.^40^, while Shannon effective count measure even distribution of taxa and can be considered as a number of dominant species. In the studies of the human microbiota, *α*-diversity is commonly used as an indicator of the state of bacterial communities and has been linked with human health^55^. Our results showed that both *α*-diversity metrics, Richness and Shannon effective count were significantly lower in IND groups compared to the non-cultured MIX inoculum which indicates that sample pooling has a favorable effect on the increase of species richness, and species evenness. Similar results have been reported by Rodríguez-Ruano et al.^48^, who showed a clear shift in *α*-diversity between the microbiome of mosquitoes processed as individuals and pooled samples. On the contrary, Aguirre et al.^30^ using Simpson’s diversity metrics showed that the pooled adult faecal inoculum resulted in comparable bacterial community composition to the individual faecal inocula. These authors performed this study on a small cohort (n = 4), and the age of healthy volunteers ranged between 29 and 62. The results of microbiome research could be affected by several factors. The differences in the bacterial community between individuals are influenced by many factors including diet, age, lifestyle, socioeconomic status, geography, and genetics^56–58^. Moreover, GM as a dynamic system has been described as unstable concerning the overall taxonomy in the first few years of life and stable during adult life^49,59,60^. The influence of these factors can make data from multiple studies difficult to compare, therefore, further studies are needed to clarify the effects of different factors on high inter-individual variability in analyzed human populations.

In our study, core microbiome analysis revealed only 17 unique ASVs common for all donor samples which indicates a high diversity among individual donors. The common core microbiome is usually defined as the most widespread microbial taxa within a host population but a universal taxonomic core rarely exists across groups of humans^29,58^. Nonetheless, the common core microbiome allows a better understanding of how microbiomes are structured across host populations and species. Moreover, the pooling of faecal samples allowed the retrieval of the majority of individual taxa represented in donor samples because 87.21% of ASVs were identified in pooled samples, and 52.27% of ASVs from individual donors constituted the pooled sample core. Similar results have also been reported by Rodríguez-Ruano et al.^48^ who showed that the core microbiome was overestimated in pooled mosquito microbiomesamples and taxa present only in a few individuals could be included in the core microbiome.

It is known that the GM community consists of many fastidious microorganisms, some of which remain non-culturable^61^, therefore we applied PMA treatment to assess the bacterial viability in pooled frozen faecal samples (MIX) which were subsequently used for inoculation of anaerobic in vitro batch cultures. Results showed that PMA-treated inoculum (MIX) was characterized by significant decrease in species richness and evenness. This finding indicates that the collection and processing of stool samples before cultivation is critical points for preserving the viability of the GM community. Bellali et al.^61,62^ reported that 28% of bacterial OTUs in the fresh faecal samples were non-viable and a large number of them represented anaerobically and not yet cultured bacteria. The viability of GM is greatly influenced by sampling and exposure to oxygen, freezing, and the time between the collection and transport to the laboratory^27,61–63^. Recent studies have shown that the non-viable bacterial content of stool processed immediately under strictly anaerobic conditions can be up to 50%^27,28^. Moreover, some taxa may remain uncultured because all members of such species are already non-viable when they reach the end of the digestive tract^61^.

However, there is a lack of literature addressing which groups of bacteria are seriously damaged after freezing and thawing. According to Murray et al.^64^, the negative effect of freezing stool samples can be associated with a decrease in gram-negative bacteria abundances. However, this negative effect was not observed during the preparation of frozen human faecal inoculum by Rose et al.^65^ and Aguirre et al.^47^. These authors observed that the viable cells in the faecal inoculum storage at − 80 °C were not affected, and the microbial diversity did not substantially differ from the fresh faecal samples.

### Effect of four culture media on GM diversity

To address the effect of various types of culture media on GM taxonomic profile, we inoculated anaerobic in vitro batch cultures with pooled frozen faecal samples (MIX). To reduce viability bias, we removed DNA from non-viable bacterial cells using PMA treatment before 16S rDNA sequencing. To our knowledge, this is the first study focused on the impact of the culture media on GM growth with the use of the PMA method before the sequencing of tested samples.

According to the previous reports^19^, our results also confirmed that the composition of the cultivation medium significantly impacts the growth and change in the taxonomic profile of the GM community during in vitro cultivation. After 24 h of cultivation, the highest community *α*-diversity in terms of Shannon effective count in comparison to the non-cultured MIX inoculum was noticed in SM and GMM. However, no significant changes in taxa richness were observed between the non-cultured MIX inoculum and all tested media groups. Moreover, in terms of *β*-diversity, CFBM showed the highest dissimilarity to the non-cultured MIX inoculum. In addition, *β*-diversity indicated significant differences in taxonomic composition between the tested media, excluding CFBM and FM. Overall, we found that CFBM and FM, which are characterized by a low nutrient concentration, were associated with decrease in GM biodiversity in comparison to the media with higher nutrient concentration, SM and GMM. The FM and CFBM were created to study the microbiome’s effects of selected substances such as prebiotics, fiber etc. on GM growth and have a limited carbohydrate content^33,34^. In our study, FM and CFBM were not enriched with any additional carbon/energy source, which could have a major impact on bacterial growth during cultivation.

In our study, we observed that SM significantly promoted the increase in abundance of the unclassified taxa from the *Lachnospiraceae* family. In the human intestine, the *Lachnospiraceae* family including *Blautia*, *Coprococcus*, *Dorea*, *Lachnospira*, *Oribacterium*, *Roseburia* and *L-Ruminococcus* are the main genera that have been found by metagenomic analysis^66^ and are associated with the ability to produce beneficial metabolites such as butyrate and other SCFAs^67,68^. SM is a widely used non-selective medium for the cultivation of obligate anaerobes with a high content of nutrients, supplemented with 5% sheep blood^69^. Blood was shown as one of the key components of the microbiological medium for high-yield cultivation of the gut microbiota^17^. Blood also contains inhibitors that can restrain the growth of certain bacteria including *Neisseria* and *Haemophilus,* however, it also includes essential growth factors such as the V factor, which are released to the medium only after blood heating^69^. In turn, at the phylum level, the CFBM was associated with the lower abundance of *Bacteroidetes,* leading to an increased *Firmicutes*/*Bacteroidetes* ratio compared to the non-cultured MIX inoculum. It is known, that decrease in *Bacteroidetes* abundance is often observed in patients with obesity^70^. Moreover, in comparison with the non-culture MIX inoculum, CFBM promoted the growth of *Bifidobacterium*, which is naturally resident in the intestine of healthy adults, and a decrease in its relative abundance has been negatively correlated with markers of low-grade inflammation^71^. In contrast, a significantly higher abundance of *Bifidobacterium* in the GMM group was reported by Yousi et al.^19^, who evaluated the effect of the Brain Heart Infusion (BHI), Gut Microbiota Medium (GMM), Fastidious Anaerobe Broth (FAB), and Bacterial Growth Medium (BGM) on GM *α*-diversity of the cultivated pooled human faecal inocula; but no comparative analysis with non-culture MIX inoculum was conducted. The most abundant microbe at the genus level in the non-cultured MIX inoculum was *Blautia* which accounted for over 10% of total sequences recovered from the pooled stool samples. *Blautia* is an obligately anaerobic, non-sporulating member of the *Firmicutes* phylum, and plays an important role in the fermentation of indigestible carbohydrates, SCFAs, and antimicrobial peptides production^72^. The decrease in the abundance of *Blautia* in CFBM and FM observed in our study may be related to the insufficient content of carbohydrates as the required energy source.

In our study, we observed the media-specific composition of biomarker microorganisms which can be associated with the nutritional composition of tested media that supported the growth of different microbial taxa. However, in all media groups, the one of most differently enriched genera compared to the non-cultured MIX inoculum was *Megasphaera*, an obligately anaerobic, Gram-negative member of the *Clostridia* family^73^. *Megasphaera* has a potentially positive impact on host health as it is a producer of SCFAs, essential amino acids, and vitamins, and it modulates the host’s immune response^73,74^. The highest abundance of *Megasphaera* was observed in the CFBM group and was 18.4-fold than in the non-cultured MIX inoculum; the lowest abundance of *Megasphaera* was found in the SM group with a 4.5-fold increase compared to the non-cultured MIX inoculum. In all tested media we observed a lower abundance of *Akkermansia*, *Prevotella*, and *Ruminococcus* which are known as mucin-degrading bacteria^75^. In our study, none of the four tested culture media contained mucin as an ingredient which could potentially lead to nutrient starvation as it is an important growth factor for numerous microorganisms, especially within the *Firmicutes* phyla^76^. Moreover, GM, FM, and CFBM promoted the growth of *Clostridium sensu stricto 1*, an opportunistic pathogen, which plays a role in the occurrence of intestinal inflammation and can be associated with decreased microbial SCFAs production^77^. Thus, the decrease in SCFAs production observed in our study may have been associated with increased *Clostridium sensu stricto 1* abundance.

In this study, to consider not only the GM structure but also the microbiome function, we utilized PICRUSt to predict the metagenome profiles based on 16S rDNA gene sequence data. The result of PICRUSt predicted an increase in relative abundances of genes associated with xenobiotic biodegradation and metabolism pathways in all media groups compared to the non-cultured MIX inoculum. KEGG level 3 analysis showed that enriched pathways were related to the degradation of organic contaminants such as aminobenzoate, atrazine, benzoate, bisphenol, dioxin, styrene, toluene, xylene, and others indicating the possibility of using them for GM in vitro cultivation in toxicomicrobiomics research. GM is an important factor affecting the metabolism of drugs and the biotransformation of other endogenous xenobiotics^78^. As nearly 3000 cytochrome P450 enzymes are present in GM genomes that are involved in the metabolism of various endogenous and exogenous chemicals^76,79^. In addition, in the FM, KEGG analysis showed a significant increase in the metabolism of drugs by the cytochrome P450 while in SM drug metabolism by other enzymes pathway was also noticed.

### Effect of culture media on GM metabolite production

The analysis of the stool microbiome is now commonly supplemented with additional analysis of microbial metabolites, such as SCFAs that play important role in maintaining host health by modulation of metabolite absorption by colonocytes, epithelial cell proliferation, host appetite signaling, enteropathogens growth reduction, and constitute an energy source for the host^80^. The main source of gut SCFAs production is microbial fermentation of complex carbohydrates such as resistant starch, hemicelluloses, cellulose, pectin, and fructans^81^. The most abundant SCFAs are acetate (60%), propionate (25%), and butyrate (15%) in the human colon, which are used as both energy sources^80^ and signaling biomolecules^82^. Bacterial cross-feeding greatly impacts the balance of SCFAs production and affects the efficient exploitation of substrate^66^. Among all SCFAs, butyrate is considered an important energy source for colonic epithelial cells, supplying approximately 60–70% of their total energy requirements^33,49,75,83^.

In our study, the production of the SCFAs, including acetic, propionic, isobutyric, butyric, valeric, and isovaleric acid by GM in four different media after 24 h in vitro cultivation was assessed. Similar to Yousi et al.^19^, we also confirmed that the composition of the cultivation medium significantly impacts the change of the microbial SCFAs profile during in vitro cultivation. The highest production of total SCFAs has been noticed for GM cultivated in the SM, whereas in both the SM and the GMM acetic acid accounted for the largest proportion of SCFAs produced. Similar results have been reported by Yousi et al.^19^, who demonstrated that GMM promoted the highest production of acetic acid among four tested groups of media. Moreover, we observed the high production of propionic acid in SM (1181.99 ± 233.50 mg/l) and GMM (1991.85 ± 249.58 mg/l) after 24 h of in vitro cultivation. Yousi et al.^19^ reported that after 48 h of in vitro incubation, propionate production in the GMM reached the level of 1240 ± 120 mg/l.

In our study, a significant decrease in *Roseburia*, *Ruminoccocus,* and *Agathobacter* abundances as important fibrinolytic SCFA producers were noticed in all tested media compared to the non-cultured MIX inoculum. We suppose that during GM cultivation, SCFA production probably took place via the utilization of sugars by the glycolytic community (e.g., *Lactobacillus*, *Enterococcus*, *Staphylococcus*) and further metabolism of the obtained cross-metabolites by the lactate-utilizing community (e.g., *Eubacterium*, *Anaerostipes*, *Propionibacterium*). Moreover, hydrogen largely produced during sugar (or fibre) fermentation is metabolized by the hydrogenotrophic community (e.g., *Blautia*) to acetate. Next, another group of microbes, including *Roseburia*, and *Eubacterium* can utilize acetate to produce butyrate as a part complex cross-feeding chain^81^. Therefore, we presume that decreased total SCFAs production observed in FM and CFBM was due to the absence of added carbohydrate sources that could be used during the cultivation process.

GM is a highly metabolically active community, and its interactions with the host metabolism are often mediated by bacterial metabolites^79,84,85^. The nutritional components in a cultivation medium are key determinants of GM metabolic performance during in vitro cultivation^86^. Nonetheless, there is still a lack of systematic studies on how medium compositions impact the metabolome of GM cultured in vitro.

In our study, the LC-HR-MS/MS approach was used to analyze the impact of cultivation media on the GM metabolome profiles. MS-based untargeted metabolomics is considered to be more sensitive than other NMR-based methods, but it commonly leads to the generation of a vast number of unidentified metabolic features^87,88^. Nevertheless, both NMR and MS-based methods are considered complementary and recommended to use in combination to obtain detailed information on the shift metabolic profiles and metabolic pathways network interactions in response to medium supplementation during in vitro culture conditions^88,89^. Our results showed that all the studied media had a unique impact on the GM metabolite profiles after 24 h of in vitro culture, but almost 80% of detected metabolites were unidentified. These results make it difficult to conclude which components in tested media can stimulate the production of specific metabolites and maintenance of the microbiota’s functional activities, but using KEGG analysis, increase the bile secretion, biosynthesis of unsaturated fatty acids, linoleic acid metabolism, biosynthesis of secondary metabolites and metabolic pathways were noticed. For example, many studies have associated bile acid metabolism (Bas) with GM composition^90–92^ and about 3% of the total number of correlations between metabolites and metabolite pathways in faeces were with Bas^92^. Nonetheless, our metabolome analysis showed that the cultivation in the GMM medium led to increased production of a considerable number of metabolites which were lowered in other media groups. Some of those metabolites were assigned to KEGG pathways connected with various forms of metabolism. It has been reported that despite the high inter-individual variability of GM, functional pathways are stable within a healthy human population^31^. For instance, Visconti et al.^92^ reported that 56% of the metabolic pathways are detected in more than 50% of the human faecal samples. In contrast, 87% of GM species are detected in less than 50% of the samples. In turn, Aguirre et al.^30^ showed a similar trend in metabolic activity in terms of SCFA production using inocula prepared from individual donors and a pool of these. Furthermore, the stool metabolome reflects the GM-host interactions, which greatly impact host biological functions and health status^93,94^. Together, these results demonstrate that the application of untargeted metabolomics for in vitro GM cultivation studies could be a powerful tool for the discovery and identification of metabolite biomarkers correlated with human diseases.

In summary, although GM in vitro cultures have limitations, it provides a non-invasive model to study the outcomes of different factors shaping the GM community and its metabolic activity, such as diet, xenobiotics, and drugs, therefore indirectly having an impact on the host health and well-being^87,95,96^.

## Conclusions

Similar to previously published reports^19,30,31,48^, our results have also shown the suitability of using the pooled faecal inoculum in the in vitro GM batch cultures. Moreover, we have presented the varying effects of different cultivation media on global human GM community composition and metabolic activity. In our study, we observed that the use of low-carbohydrate media (FM, CFBM) led to a significant decrease of GM biodiversity and SCFAs production. The commercial Schaedler broth (SM) supplemented with 5% sheep blood preserved the high biodiversity and metabolic activity of the human GM. Moreover, the significant enrichment of the biodegradation pathways of xenobiotics observed in the SM medium indicates its usefulness in future studies on the impact of exogenous xenobiotics on the human intestinal microbiota. Our results indicate that the limitation of the study was the inability to cultivate the whole GM population in any tested media. However, a considerable proportion of the bacterial community within the GM can be preserved in a single-growth medium. Appropriate supplementation of medium is essential for the growth of fastidious taxa. It should be noted that the interpretation of results obtained in our study was restricted by the small number of samples as well as utilizing of the 16S rDNA amplicon sequencing approach^97^. Nonetheless, we believed that these results can provide useful insight to improve the GM in the in vitro cultivation process.

## Supplementary Information

Supplementary Information.

## Data Availability

The datasets generated during the current study are available in the GeneBank NCBI repository, Bioproject ID: PRJNA875273 (http://www.ncbi.nlm.nih.gov/bioproject/875273).
